# CDK12/13 inactivation triggers STING-mediated antitumor immunity in preclinical models

**DOI:** 10.1172/JCI193745

**Published:** 2025-07-22

**Authors:** Yi Bao, Yu Chang, Jean Ching-Yi Tien, Gabriel Cruz, Fan Yang, Rahul Mannan, Somnath Mahapatra, Radha Paturu, Xuhong Cao, Fengyun Su, Rui Wang, Yuping Zhang, Mahnoor Gondal, Jae Eun Choi, Jonathan K. Gurkan, Stephanie J. Miner, Dan R. Robinson, Yi-Mi Wu, Licheng Zhou, Zhen Wang, Ilona Kryczek, Xiaoju Wang, Marcin Cieslik, Yuanyuan Qiao, Alexander Tsodikov, Weiping Zou, Ke Ding, Arul M. Chinnaiyan

**Affiliations:** 1 Michigan Center for Translational Pathology,; 2 Department of Pathology, and; 3 Department of Computational Medicine and Bioinformatics, University of Michigan, Ann Arbor, Michigan, USA.; 4 State Key Laboratory of Chemical Biology, Shanghai Institute of Organic Chemistry, Chinese Academy of Sciences, Shanghai, China.; 5 International Cooperative Laboratory of Traditional Chinese Medicine Modernization and Innovative Drug Discovery of Chinese Ministry of Education (MOE), Guangzhou City Key Laboratory of Precision Chemical Drug Development, College of Pharmacy, Jinan University, Guangzhou, China.; 6 Department of Surgery,; 7 Center of Excellence for Cancer Immunology and Immunotherapy,; 8 Rogel Cancer Center,; 9 Department of Biostatistics,; 10 Howard Hughes Medical Institute, and; 11 Department of Urology, University of Michigan, Ann Arbor, Michigan, USA.

**Keywords:** Cell biology, Oncology, Cancer, Cancer immunotherapy, Therapeutics

## Abstract

Inactivation of cyclin-dependent kinase 12 (CDK12) defines an immunogenic molecular subtype of prostate cancer characterized by genomic instability and increased intratumoral T cell infiltration. This study revealed that genetic or pharmacologic inactivation of CDK12 and its paralog CDK13 robustly activates stimulator of interferon genes (STING) signaling across multiple cancer types. Clinical cohort analysis showed that reduced CDK12/13 expression correlates with improved survival and response to immune checkpoint blockade (ICB). Mechanistically, CDK12/13 depletion or targeted degradation induced cytosolic nucleic acid release, triggering STING pathway activation. CDK12/13 degradation delayed tumor growth and synergized with anti–PD-1 therapy in syngeneic tumor models, enhancing STING activity and promoting CD8^+^ T cell infiltration and activation within tumors. Notably, the antitumor effects of this combination required STING signaling and functional CD8^+^ T cells. These findings establish STING activation as the key driver of T cell infiltration and the immune-hot tumor microenvironment in CDK12-mutant cancers, suggesting that dual CDK12/13 inhibitors and degraders activate antitumor immunity and potentiate responses to immunotherapies.

## Abstract 2

Blocking CDK12/13 boosts immune response against cancer by activating STING, helping immunotherapy work better—offering a new way to treat more patients effectively.

## Introduction

The interplay between tumor biology and the immune response has emerged as a crucial area of research in cancer therapy, particularly in the context of immune checkpoint blockade (ICB) (1, 2). Nevertheless, a substantial proportion of patients experience resistance to ICB, either through intrinsic mechanisms within the tumor microenvironment or through acquired resistance during therapy (2, 3). Accumulating studies have demonstrated that the stimulator of interferon (IFN) genes (STING) signaling pathway, which can be activated by cytosolic nucleic acids (4–6), plays a key role in boosting the immune response against tumors and enhancing ICB efficacy (5–10). Activation of STING is known to promote the expression of IFN-stimulated genes, including multiple chemokines that recruit CD8^+^ T cells, which are essential for effective antitumor immunity, as well as MHC class I (MHC-I) genes that are crucial for facilitating tumor cell recognition by CD8^+^ T cells (8, 9, 11, 12).

Cyclin-dependent kinase 12 (CDK12) and its paralog CDK13 phosphorylate RNA polymerase II (RNAPII) to mediate proper transcriptional elongation and processivity (13–16). In advanced prostate cancer and ovarian cancer, inactivating mutations of *CDK12* define distinct molecular subtypes of the disease (17, 18). *CDK12* inactivation has been linked to higher levels of intratumoral T cells in advanced prostate cancer (17, 19). Previously, we reported that *Cdk12* ablation in the murine prostate epithelium induces preneoplastic lesions with lymphocytic infiltration, and a paralog synthetic lethal relationship exists between CDK12 and CDK13 (hereafter referred to as CDK12/13) (13). However, the mechanisms underlying these phenotypes and their therapeutic implications remained unclear. Given that biallelic loss of *CDK12* represents an immunogenic molecular subtype of prostate cancer and the crucial role of T cells in antitumor immunity, we speculated that CDK12/13 could represent a therapeutic avenue for triggering antitumor immune responses and thereby enhance ICB efficacy.

In this study, we found that attenuated expression of *CDK12/13* was associated with STING activation and an improved response to ICB. Using an orally bioavailable proteolysis-targeting chimera (PROTAC) degrader of CDK12/13, YJ1206 (20), we further investigated the mechanisms linking CDK12/13 expression to STING activation. We discovered that pharmacological degradation or genetic depletion of *CDK12/13* led to the formation of transcription-replication conflicts (TRCs) and R-loops, which in turn caused upregulation of cytosolic DNA and activation of STING signaling. Notably, YJ1206 treatment not only activated STING signaling in vivo but also significantly synergized with anti–PD-1 therapy to suppress tumor growth. Thus, this study provides the first evidence to our knowledge that *CDK12* mutation triggers activation of the cyclic GMP-AMP synthase (cGAS)/STING pathway, a key component of antitumor immunity, and establishes STING activation as the underlying mechanism driving T cell recruitment in cancers with *CDK12* mutations. Our findings suggest that CDK12/13 antagonists activate STING signaling and may be leveraged to overcome resistance to immunotherapies, particularly ICB, addressing a critical clinical challenge in cancer treatment.

## Results

### CDK12/13 inactivation or attenuated expression levels are associated with elevated STING activation and improved response to ICB.

**Figure 1:**
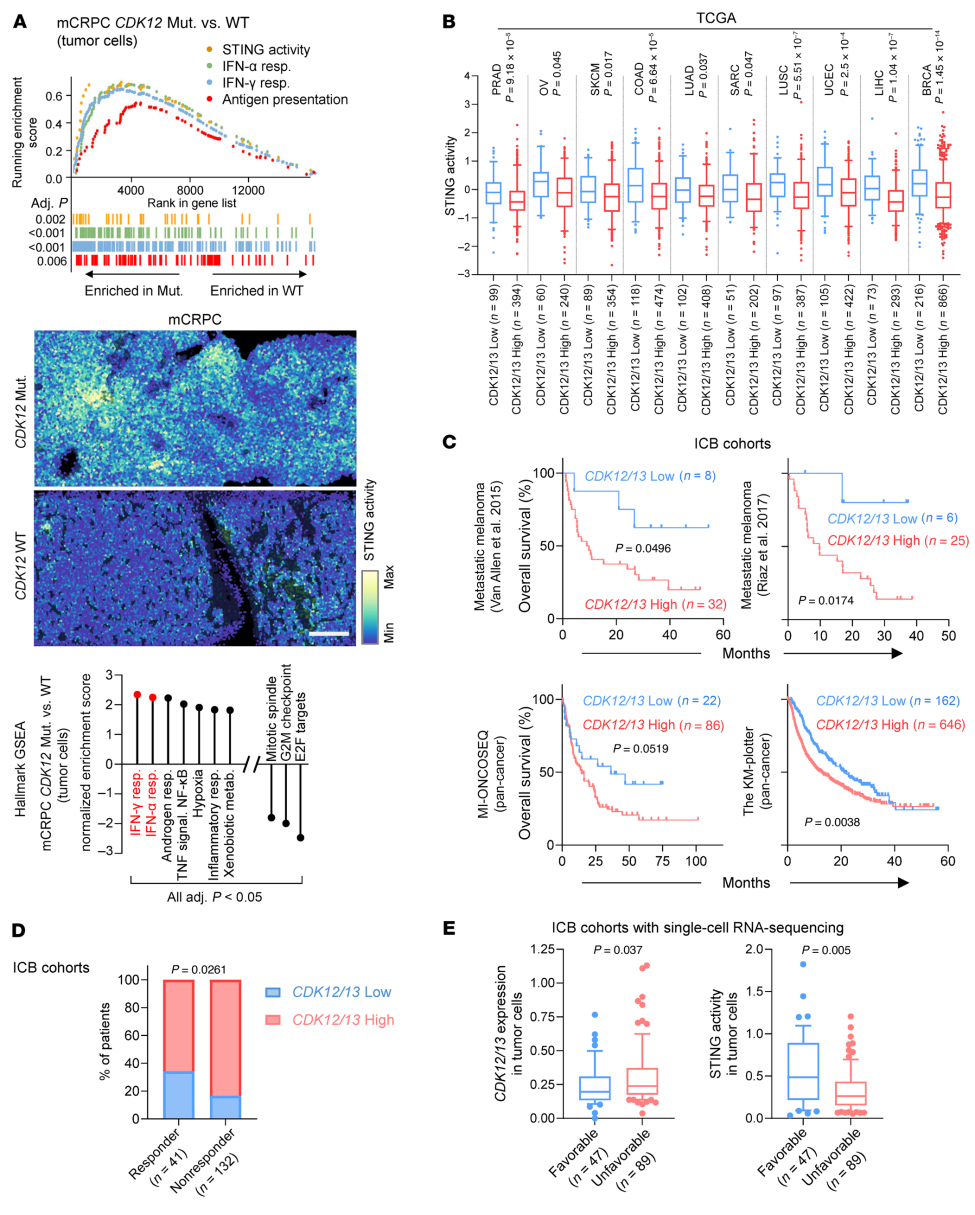
*CDK12/13* inactivation or low expression levels are associated with elevated STING activation and improved response to ICB. (**A**) Analyses of 10× Genomics Visium spatial transcriptomics on *CDK12* mutant versus wild-type (WT) metastatic castration-resistant prostate cancer (mCRPC) samples. Top: Enrichment of the indicated pathways in tumor cells. Middle: Representative images illustrating the enrichment of STING activity signature in tumor areas. Bottom: The top 10 pathways enriched by gene set enrichment analysis (GSEA), utilizing the MSigDB Hallmark database, in tumor cells. Type I and II IFN responses are highlighted in red. Adj., adjusted. Scale bar: 200 μm. (**B**) Association between expression of *CDK12/13* and STING activity signature in the indicated cancer types. Data were acquired from The Cancer Genome Atlas datasets. Abbreviations are defined in the legend of Supplemental Figure 1. (**C**) Association between pretreatment expression of *CDK12/13* and overall survival in the indicated cohorts treated with ICB therapy. MI-ONCOSEQ: a pan-cancer cohort at the University of Michigan. The KM plotter data were acquired from the KM plotter database (https://kmplot.com/analysis/). (**D**) Association between pretreatment expression of *CDK12/13* and ICB response in the melanoma and MI-ONCOSEQ ICB cohorts in **C**. (**E**) Single-cell RNA sequencing assessing expression of indicated genes or signature in tumor cells in ICB-treated cohorts. Left: Expression of *CDK12/13* in tumor cells in patients with favorable versus unfavorable clinical outcomes. Right: Expression of STING activity signature in tumor cells in patients with favorable versus unfavorable clinical outcomes. Data were acquired from published research articles (see Methods). Low expression of *CDK12/13* was defined as the bottom 20th percentile within each cohort. Two-tailed *t* tests were performed in **B** (with Bonferroni’s correction) and **E**, log-rank tests in **C**, and Fisher’s exact test in **D**. Panels **B** and **D** show box-and-whisker plots with the median (center line), 25th–75th percentiles (box), 10th–90th percentiles (whiskers), and outliers beyond the whiskers.

We previously reported that inactivating mutations in *CDK12* were associated with higher levels of intratumoral T cells in advanced prostate cancer (17). To understand the underlying mechanisms, we performed spatial transcriptomic analyses on metastatic castration-resistant prostate cancer (mCRPC) samples with either inactivated or wild-type *CDK12*. We found that inactivation of *CDK12* was significantly associated with a tumor-specific enrichment of STING activity signature (8) (Figure 1A), along with signatures primarily composed of IFN-stimulated genes that could be induced by STING activation (8) (Figure 1A). These signatures included type I (IFN-α) and II (IFN-γ) IFN responses and antigen presentation (Figure 1A). Additionally, gene set enrichment analysis (GSEA) utilizing the MSigDB Hallmark database revealed that type I and II IFN responses were the most upregulated pathways in *CDK12*-inactivated tumors compared with the wild-type (Figure 1A). Mutations of *CDK12* are rare in cancers, other than ovarian cancer and advanced prostate cancer. However, low expression of *CDK12* was significantly associated with increased STING activity in various cancer types (Supplemental Figure 1A, top; supplemental material available online with this article; https://doi.org/10.1172/JCI193745DS1). CDK13, a paralog of CDK12, has been shown to have functional redundancy with CDK12 (13, 14, 16). Notably, low expression of *CDK13* also tended to be associated with activated STING signaling (Supplemental Figure 1A, bottom). Importantly, the combined low expression of both *CDK12* and *CDK13* exhibited the strongest association with elevated STING activity (Figure 1B), along with significant upregulation of IFN response (Supplemental Figure 1B). Therefore, attenuated expression levels of *CDK12/13* may lead to the activation of STING signaling in various cancer types.

Activation of STING signaling has been shown to enhance the efficacy of ICB therapy (9, 21). Given the strong association between STING activity and *CDK12/13* expression, we examined whether low expression of *CDK12/13* predicted survival in patients treated with ICB therapy. We first analyzed RNA sequencing data from tumor samples collected prior to ICB treatment in 2 metastatic melanoma cohorts (22, 23) and found that low expression of *CDK12/13* (bottom 20th percentile) was significantly associated with improved overall survival (Figure 1C). We next evaluated the predictive value of *CDK12/13* expression in a pan-cancer setting. In the pan-cancer ICB cohort from the University of Michigan (MI-ONCOSEQ; *n* = 108; Supplemental Table 1), low pretreatment expression of *CDK12/13* (bottom 20th percentile) was similarly associated with improved survival, reaching borderline statistical significance (*P* = 0.0519; Figure 1C), suggesting that a larger sample size may be required to achieve statistical significance. To further validate this association, we analyzed data from the Kaplan-Meier (KM) plotter database (https://kmplot.com/analysis/) (24, 25), which comprises a substantially larger ICB-treated pan-cancer population (*n* = 808; Supplemental Table 2). As expected, low pretreatment *CDK12/13* expression (bottom 20th percentile) was significantly associated with improved survival (*P* = 0.0038; Figure 1C). Collectively, these findings support low *CDK12/13* expression as a predictive biomarker for improved overall survival in ICB-treated patients.

Additionally, low expression of *CDK12/13* significantly predicted response to ICB therapy (Figure 1D). Notably, multiple linear regression analysis revealed that low pretreatment *CDK12/13* expression (Supplemental Figure 2, left) or high pretreatment STING activity signature expression (Supplemental Figure 2, right) was independently associated with improved clinical outcomes, after adjusting for age, sex, and tumor site. We further explored single-cell sequencing data from ICB-treated cohorts to specifically evaluate the expression of *CDK12/13* in tumor cells (Figure 1E). As anticipated, we observed that reduced expression of *CDK12/13* (Figure 1E) or elevated STING activity (Figure 1E) was significantly associated with favorable clinical outcomes in these cohorts. Collectively, these findings highlight that low expression of *CDK12/13* is predictive of better clinical outcomes in cohorts treated with ICB.

### Targeting CDK12/13 activates STING signaling.

**Figure 2:**
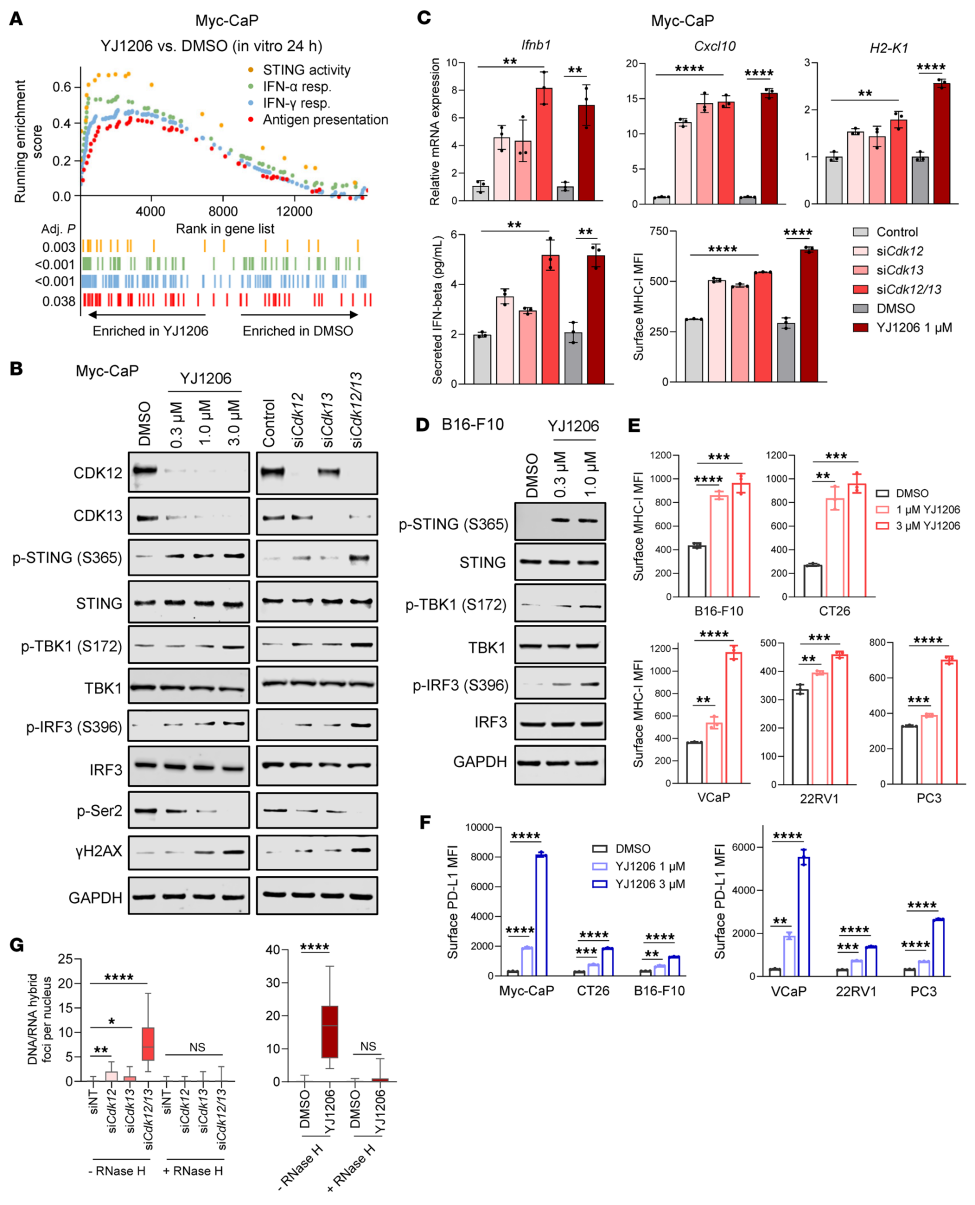
Targeting CDK12/13 activates STING signaling. (**A**) Enrichment of the indicated pathways in Myc-CaP cells treated with YJ1206 at 1 μM for 24 hours. Adj., adjusted. (**B**) Immunoblot of the indicated proteins in Myc-CaP cells treated with YJ1206 at increasing concentrations for 4 hours, or siRNAs targeting *Cdk12* and/or *Cdk13*. Nontargeting siRNA was used as control. GAPDH was used as a loading control. (**C**) Top: Analysis of the indicated gene expression by RT-qPCR in Myc-CaP cells treated with YJ1206 at 1 μM for 15 hours, or siRNAs targeting *Cdk12* and/or *Cdk13*. Nontargeting siRNA was used as control. Bottom left: IFN-β ELISA results in Myc-CaP cells treated as described above. Bottom right: Flow cytometry assessing surface MHC-I expression in Myc-CaP cells treated as described above. (**D**) Immunoblot of the noted proteins in B16-F10 cells treated with YJ1206 at increasing concentrations for 4 hours. (**E** and **F**) Flow cytometry median fluorescence intensity (MFI) quantifications of surface MHC-I (**E**) or PD-L1 (**F**) in the indicated cells treated with YJ1206 at 1 μM or 3 μM for 15 hours. (**G**) Quantification of immunofluorescence DNA/RNA hybrid (red) staining in Myc-CaP cells treated with 1 μM YJ1206 for 4 hours or siRNA targeting *Cdk12* and/or *Cdk13*, with/without RNase H. Representative images are in Supplemental Figure 4D. Nontargeting siRNA was used as control. Forty (siRNA treatment) or 20 (YJ1206 or DMSO treatment) cells were used per data point. Data in **C**, **E**, and **F** are displayed as mean ± SD of triplicate experiments. Data in **G** are presented as box-and-whisker plots, with the median (center line), 25th–75th percentiles (box), and minimum to maximum values (whiskers). *P < 0.05; **P < 0.01; ***P < 0.001; ****P < 0.0001 by 2-tailed *t* test. NS, not significant. Bonferroni’s correction was applied for multiple comparisons in **C** and **E**–**G**.

We next sought to determine whether targeting CDK12/13 could activate STING signaling. We previously developed a highly selective dual CDK12/13 PROTAC degrader, YJ1206, which is orally bioavailable and shows a favorable safety profile (20). In a murine prostate cancer model, Myc-CaP, we treated cells with YJ1206 and subsequently performed RNA sequencing analyses. The results revealed that the STING activity signature, as well as signatures that could be induced by STING activation (8), were significantly enriched in YJ1206-treated cells compared with controls (Figure 2A and Supplemental Figure 3A). Immunoblotting confirmed that treatment with YJ1206 markedly activated STING signaling, as evidenced by the increased levels of phospho-STING (p-STING), phospho-TBK1 (p-TBK1), and phospho-IRF3 (p-IRF3) (Figure 2B). Importantly, treatment with YJ1206 efficiently degraded CDK12/13, leading to a decrease in phosphorylation of serine 2 (Ser2) on RNAPII and an increase in γH2AX (Figure 2B and Supplemental Figure 3B), consistent with previous findings (20). To verify that the effect of YJ1206 was on target, we depleted *CDK12/13* using gene-specific siRNAs. As expected, genetic depletion of *CDK12/13* also resulted in STING signaling activation, decreased p-Ser2 levels, and increased γH2AX expression (Figure 2B). The activation of STING signaling was further confirmed by the elevated expression of its downstream targets, such as *Ifnb1*, *Cxcl10*, and *Ccl5* (Figure 2C and Supplemental Figure 3C), as well as genes responsive to type I IFN, *H2-K1* (an MHC-I gene; Figure 2C) and *Cd274* (which encodes PD-L1; Supplemental Figure 3C). Importantly, similar findings were observed in additional murine cancer models, B16-F10 (melanoma) and CT26 (colon carcinoma) (Figure 2, D–F), as well as across a panel of human cancer cell lines (Figure 2, E and F, and Supplemental Figure 3D). This indicates that the phenotype was not model or strain specific. Of note, inhibition of many other oncotherapeutic targets, including the BET family of bromodomain proteins, ubiquitin-like modifier activating enzyme 1, mSWI/SNF ATPases, GSPT1, and CBP/p300, did not activate STING signaling, suggesting a distinct role of CDK12/13 degradation in activating this pathway (Supplemental Figure 3E). Moreover, inhibition of CDK12/13 with a selective CDK12/13 inhibitor, YJ5118, induced STING activity at a level comparable to that observed with the CDK12/13 degrader (Supplemental Figure 3, F and G). Collectively, these findings show that targeting CDK12/13 activates STING signaling in tumor cells.

Activation of the STING pathway may lead to activation of NF-κB signaling (5), which may upregulate MHC-I expression (26, 27). To identify key components involved in MHC-I upregulation via CDK12/13 antagonism, we knocked out *Cgas*, *Sting1* (encoding STING), *Ifnar1* (encoding IFNAR1), or *Rela* (encoding p65; Supplemental Figure 4A) in cancer cell lines. As anticipated, knockout (KO) of *Cgas*, *Sting1*, or *Ifnar1* abolished MHC-I induction by CDK12/13 antagonism in various cancer models (Supplemental Figure 4B). Interestingly, while *Rela* KO significantly reduced MHC-I expression — consistent with its known role as an MHC-I regulator — CDK12/13 antagonism still significantly induced MHC-I expression in *Rela*-KO cells (Supplemental Figure 4C). These findings suggest that the cGAS/STING pathway and type I IFN response are crucial for MHC-I upregulation mediated by CDK12/13 antagonism, whereas NF-κB signaling is not essential.

Transcription replication conflicts (TRCs) have been linked to loss of the transcription elongation kinase CDK12 (13). R-loops (RNA-DNA hybrids) formed as a result of TRCs have been attributed to triggering the release of cytosolic DNAs, which activate STING signaling via cGAS (28, 29). Therefore, we hypothesized that genetic depletion or pharmacological degradation of CDK12/13 may lead to R-loop formation. We, thus, depleted *Cdk12*, *Cdk13*, or both in tumor cells with siRNAs and assessed R-loops with the S9.6 antibody (30, 31), the specificity of which was confirmed using RNase H treatment (Figure 2G and Supplemental Figure 4D). *Cdk12* depletion resulted in increased R-loop formation (Figure 2G and Supplemental Figure 4D). Similarly, depletion of *Cdk13*, the paralog of *Cdk12*, also led to elevated R-loop levels (Figure 2G and Supplemental Figure 4D). Notably, simultaneous depletion of both *Cdk12* and *Cdk13* resulted in the highest increase in R-loop formation (Figure 2G and Supplemental Figure 4D). This observation was confirmed through treatment with the CDK12/13 degrader, YJ1206 (Figure 2G and Supplemental Figure 4D). Thus, genetic depletion or pharmacological degradation of CDK12/13 gives rise to a drastic increase in R-loop formation.

### STING activation induced by CDK12/13 inactivation is TRC dependent.

**Figure 3:**
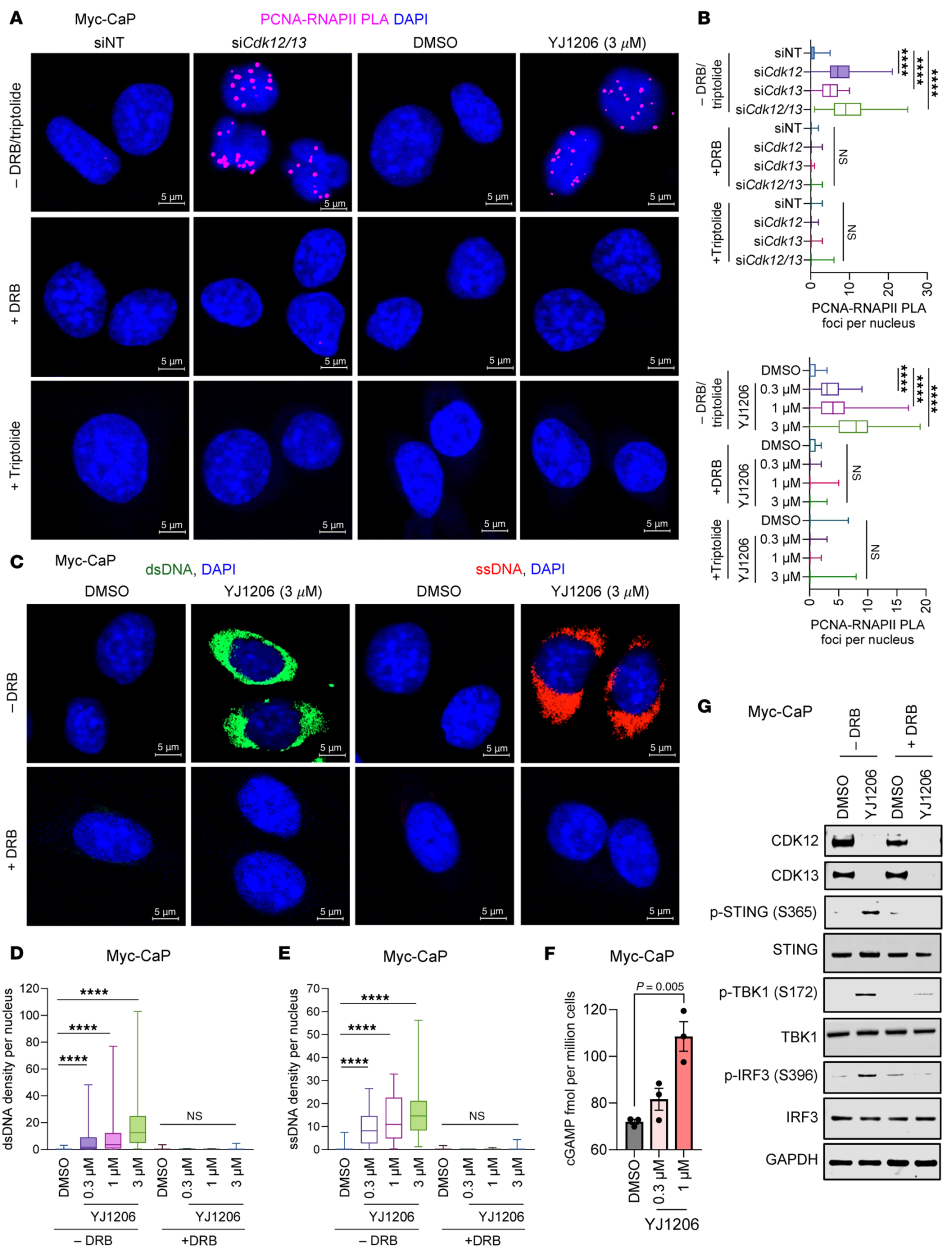
STING activation induced by CDK12/13 inactivation is TRC dependent. (**A** and **B**) Representative images (**A**) or quantification (**B**) of immunofluorescence assessing PCNA-RNAPII PLA foci in Myc-CaP cells treated with siRNA targeting *Cdk12* and/or *Cdk13*, or YJ1206 at 3 μM for 4 hours, with or without DRB or triptolide treatment. (**C**–**E**) Representative images (**C**) or quantification (**D** and **E**) of dsDNA (**C**, left, and **D**) and ssDNA (**C**, right, and **E**) in Myc-CaP cells treated with 3 μM YJ1206 for 4 hours, with or without DRB treatment. Scale bars: 5 μm. (**F**) ELISA measuring cGAMP levels in Myc-CaP cells treated with YJ1206 at the indicated concentrations. (**G**) Immunoblot of the noted proteins in Myc-CaP cells treated with YJ1206 at 1 μM, with or without DRB for 4 hours. Data in **D** and **E** are presented as box-and-whisker plots, with the median (center line), 25th–75th percentiles (box), and minimum to maximum values (whiskers). Data are displayed as mean ± SEM in **F** of triplicate experiments. One hundred cells were used per data point in **B**, **D**, and **E**. ****P < 0.0001 by 2-tailed *t* test. NS, not significant. Bonferroni’s correction was applied for multiple comparisons.

We next examined whether CDK12/13 degradation also resulted in formation of TRCs, using the proximity ligation assay (PLA) to detect the physical proximity of proliferating cell nuclear antigen (PCNA) to RNAPII (32). We observed that depletion of either *Cdk12* or *Cdk13* yielded a relatively moderate increase in TRC formation (Figure 3, A and B, and Supplemental Figure 5A). In contrast, simultaneous depletion of both *Cdk12* and *Cdk13* led to a drastic increase in TRC formation (Figure 3, A and B). This was further validated with the dual CDK12/13 PROTAC degrader (Figure 3, A and B) or depleting *Cdk12/13* using CRISPR-Cas9 (Supplemental Figure 5B). The specificity of the PLA was confirmed with 5,6-dichloro-1-β-D-ribofuranosyl-benzimidazole (DRB) or triptolide treatment, which blocked transcription (32) (Figure 3, A and B). Collectively, our findings indicate that depletion or degradation of CDK12/13 leads to TRC formation.

Head-on TRCs, whereby the transcription and replication machinery move toward each other, are known to promote R-loop formation (33, 34), which can contribute to genomic instability (34–36). We treated cells in which CDK12/13 were degraded with DRB, which abolished TRC formation (Figure 3, A and B), and measured the R-loop levels. As anticipated, DRB treatment also eliminated the increased R-loop levels resulting from CDK12/13 degradation (Supplemental Figure 5, C and D). To establish the causal link between TRCs and R-loop formation, we further inhibited DNA replication using aphidicolin (37) or hydroxyurea (38). As anticipated, treatment with aphidicolin or hydroxyurea abolished TRCs induced by CDK12/13 antagonism (Supplemental Figure 6A). Notably, R-loop accumulation triggered by CDK12/13 antagonism was also strongly diminished under these conditions (Supplemental Figure 6A). These findings support a model in which CDK12/13 antagonism promotes R-loop formation secondary to TRC induction.

R-loops have been found to induce the release of cytosolic ssDNAs (29) and dsDNAs (28) to activate STING via 2′3′-cGAMP (cGAMP) production (28). As expected, CDK12/13 degradation resulted in elevated levels of both cytosolic dsDNAs and ssDNAs (Figure 3, C–E, and Supplemental Figure 6B), as well as increased cGAMP production (Figure 3F). In contrast, elevation in cytosolic dsRNAs was not detected upon CDK12/13 degradation (Supplemental Figure 6B, bottom). Importantly, DRB treatment, which abolished TRC formation (Figure 3, A and B), fully eliminated the increase in cytosolic DNAs and, thus, reversed the YJ1206-mediated activation of STING signaling (Figure 3G). Taken together, these data show that targeting of CDK12/13 induces TRCs and R-loop formation, which in turn leads to the release of cytosolic DNAs and production of cGAMP, activating STING signaling.

### CDK12/13 inactivation activates antitumor immunity and enhances response to ICB.

**Figure 4:**
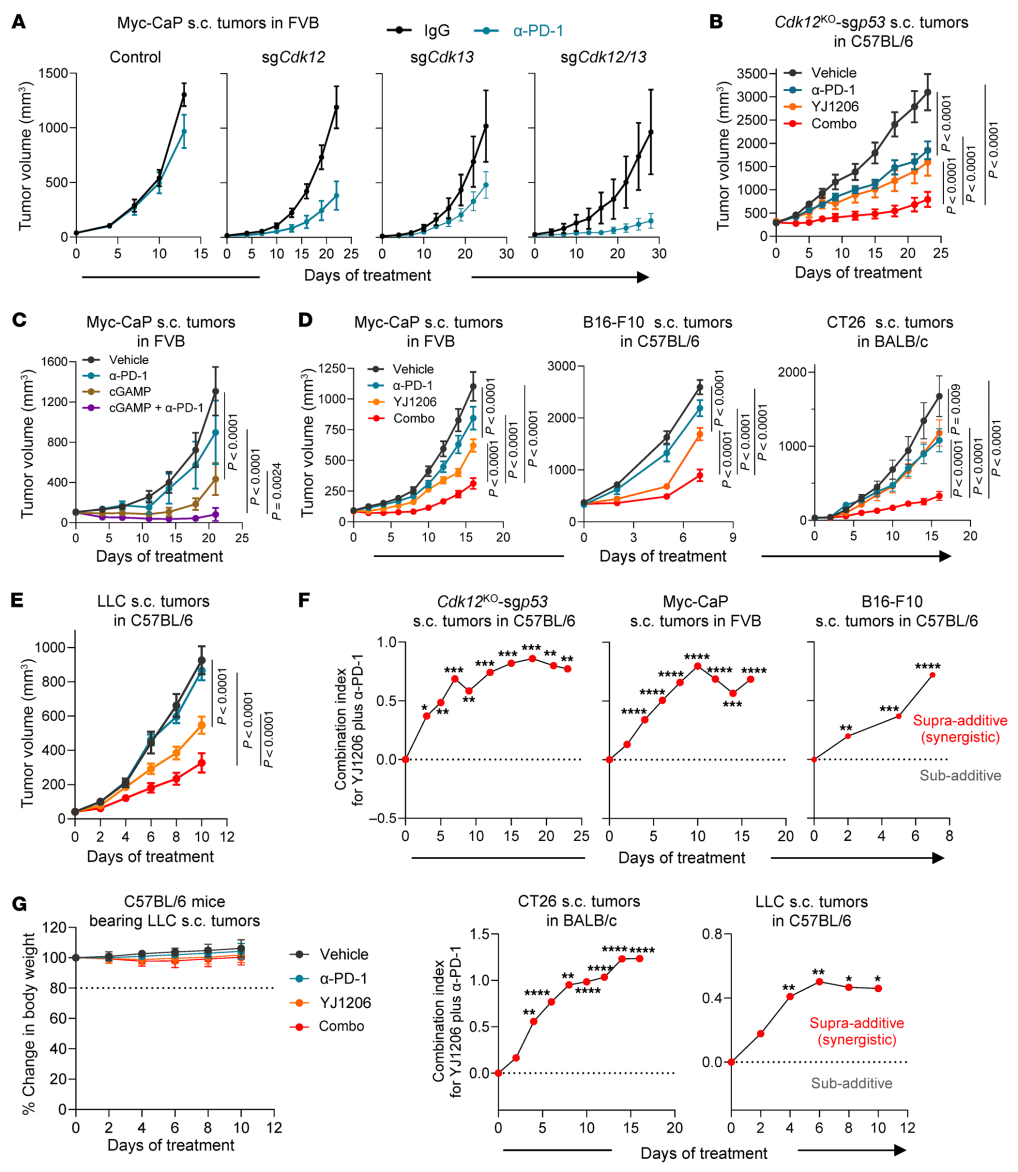
CDK12/13 inactivation activates antitumor immunity and enhances response to ICB. (**A**) Growth curves of subcutaneous (s.c.) tumors derived from Myc-CaP cells with or without *Cdk12*, *Cdk13*, or *Cdk12/13* depletion in FVB mice (*n* = 4–5 mice per group) treated with IgG or anti–PD-1 (α-PD-1). (**B**) Growth curves of s.c. tumors derived from the *Cdk12*^KO^-sg*p53* tumor cells in C57BL/6 mice (*n* = 5–6 mice per group) treated with vehicle, α-PD-1, YJ1206, or the combination of α-PD-1 and YJ1206 (combo). (**C**) Growth curves of s.c. tumors derived from Myc-CaP cells in FVB mice (*n* = 5–6 mice per group) treated with vehicle, α-PD-1, cGAMP, or the combination of α-PD-1 and cGAMP (combo). Intratumoral injections were performed for cGAMP administration. (**D** and **E**) Growth curves of s.c. tumors derived from the specified tumor cells in the indicated mice (*n* = 5–10 mice per group) treated with vehicle, α-PD-1, YJ1206, or the combination of α-PD-1 and YJ1206 (combo). (**F**) Assessment by CombPDX for synergism of α-PD-1 and YJ1206 in the indicated models treated in **B**, **D**, and **E**. (**G**) Body weight of the indicated mice after treatment by the indicated agents. YJ1206 was administered orally at a dose of 100 mg/kg, 3 times per week, and α-PD-1 was administered intraperitoneally at a dose of 200 μg/mouse every 3 days. Data are displayed as mean ± SEM in **A**–**E** and as mean ± SD in **G**. Significance in **B**–**E** was determined by 2-way ANOVA. *P < 0.05, **P < 0.01, ***P < 0.001, ****P < 0.0001. Bonferroni’s correction was applied for multiple comparisons.

Given that activation of STING enhances the efficacy of ICB therapy (9, 21), we next examined whether inactivating CDK12/13 could improve response to ICB. We depleted *Cdk12*, *Cdk13*, or both in the Myc-CaP tumor model (Supplemental Figure 7A, top). Notably, simultaneous depletion of both *Cdk12* and *Cdk13* led to the most profound improvement in response to anti–PD-1 treatment (Figure 4A and Supplemental Figure 7A, bottom), while depletion of either *Cdk12* or *Cdk13* alone led to relatively moderate enhancement of anti–PD-1 efficacy (Figure 4A). Furthermore, *Cdk12/13* depletion significantly delayed tumor growth (Supplemental Figure 7A, bottom), extending the time to endpoint from 13 days in controls to 29 days after treatment (Figure 4A). To confirm that the tumor inhibitory effect was immune dependent, we evaluated tumor growth in both immunodeficient and immunocompetent mice. Although *Cdk12/13* depletion exerted a direct tumor inhibitory effect in immunodeficient mice (Supplemental Figure 7B), consistent with previous studies (13), the tumor growth delay was significantly more pronounced in immunocompetent mice (Supplemental Figure 7B). These findings indicate that an intact immune system is essential for the full antitumor response mediated by *Cdk12/13* depletion.

The necessity of targeting both CDK12 and CDK13 to achieve optimal enhancement of ICB response was further validated with an additional prostate cancer syngeneic model harboring *Cdk12* loss and *Trp53* depletion (*Cdk12*^KO^-sg*p53*), which was previously established (13). We found that beyond the genetic loss of *Cdk12*, degradation of CDK13 by YJ1206 treatment (Supplemental Figure 7C) demonstrated antitumor effects and strongly improved ICB efficacy (Figure 4B). We next evaluated the combination of YJ1206 and anti–PD-1 across various syngeneic models, including Myc-CaP (prostate cancer), B16-F10 (melanoma), CT26 (colon cancer), and LLC (lung cancer), after confirming that activation of STING by intratumoral administration of cGAMP led to strong suppression of tumor growth and enhanced ICB efficacy (Figure 4C). We observed that degradation of CDK12/13 by YJ1206 (Supplemental Figure 7D) displayed antitumor effects and significantly improved anti–PD-1 efficacy in these models, which are otherwise insensitive to anti–PD-1 monotherapy (Figure 4, D and E, and Supplemental Figure 7E). Importantly, combination treatment with YJ1206 and anti–PD-1 demonstrated a strong synergistic effect in all models tested (Figure 4F). Consistent with the reported favorable safety profile (20), we observed no body weight loss in mice treated with YJ1206 or the combination of YJ1206 and anti–PD-1 (Figure 4G). Administration of high-dose YJ1206 also led to significant tumor suppression in immunodeficient mice (Supplemental Figure 8A), accompanied by a marked increase in tumor cell apoptosis (Supplemental Figure 8B), consistent with the literature (20). Nevertheless, in line with the *Cdk12/13* depletion data (Supplemental Figure 7B), YJ1206 treatment exhibited substantially stronger control of tumor growth in immunocompetent mice compared with immunodeficient mice (Supplemental Figure 8A). Together, our data show that targeting CDK12/13 delays tumor growth and enhances response to anti–PD-1 across various preclinical models.

### Antitumor activity of CDK12/13 degradation is CD8^+^ T cell and STING dependent.

**Figure 5:**
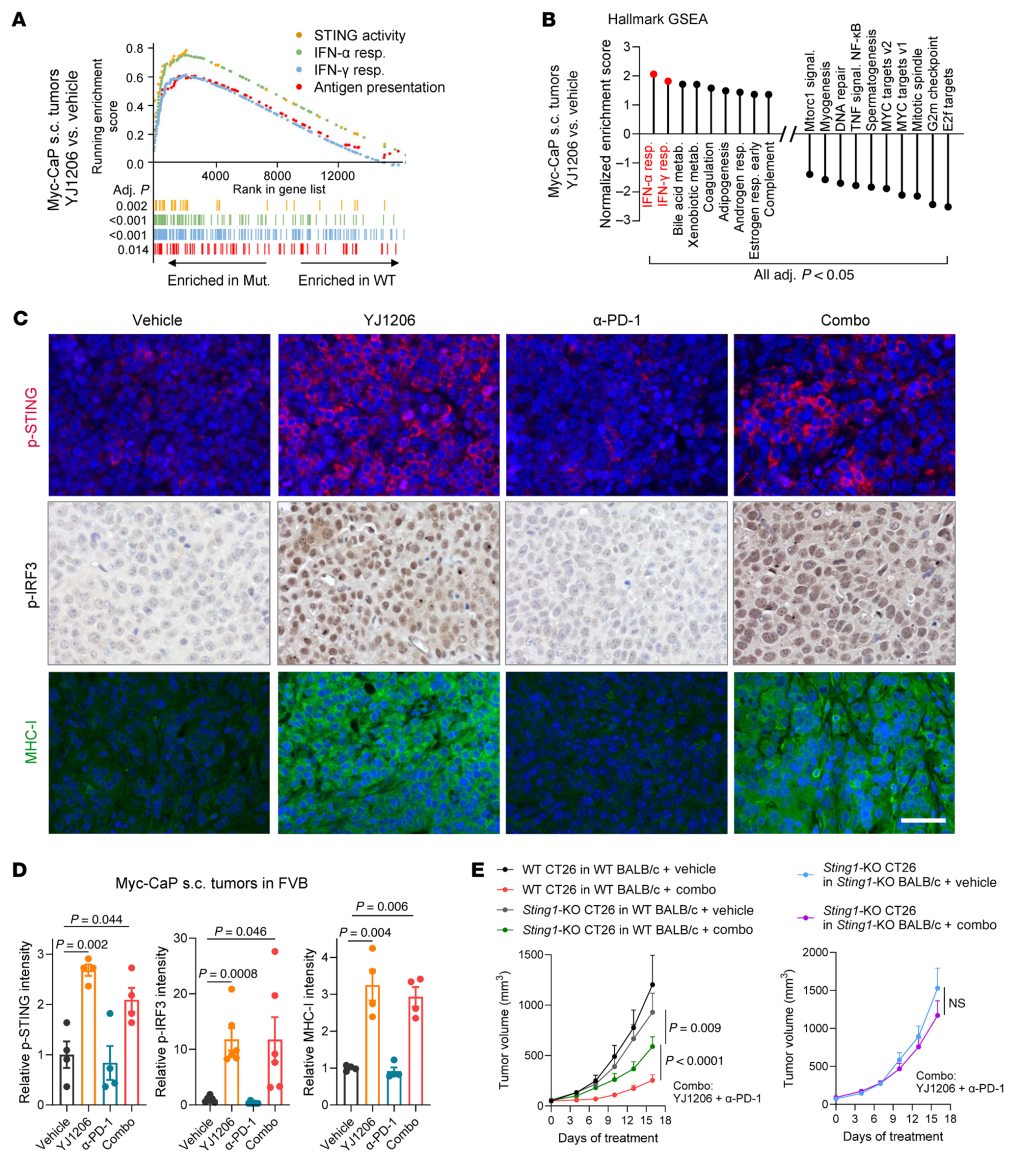
Antitumor activity of CDK12/13 degradation is STING dependent. (**A**) Enrichment of the indicated pathways in Myc-CaP subcutaneous (s.c.) tumors from FVB mice treated with YJ1206 compared to vehicle control. Adj., adjusted. (**B**) All pathways significantly enriched by GSEA, utilizing the MSigDB Hallmark database, in Myc-CaP s.c. tumors from FVB mice treated with YJ1206 compared to vehicle control. Type I and II IFN responses are highlighted in red. Adj., adjusted. (**C** and **D**) Representative images (**C**) or quantification (**D**) of immunofluorescence (for p-STING and MHC-I) or immunohistochemistry (for p-IRF3) performed on Myc-CaP tumor tissues from mice treated with vehicle, anti–PD-1 (α-PD-1), YJ1206, or the combination (combo) of α-PD-1 and YJ1206 (*n* = 4 mice per group). Scale bar: 40 μm. (**E**) Left: Growth curves of s.c. tumors derived from CT26 cells with or without *Sting1* KO, in BALB/c mice treated with the combination (combo) of α-PD-1 and YJ1206. Right: Growth curves of s.c. tumors derived from CT26 cells with *Sting1* KO, in *Sting1*-KO BALB/c mice treated with the combination (combo) of α-PD-1 and YJ1206. YJ1206 was administered orally at a dose of 25 mg/kg, 3 times per week, and α-PD-1 was administered intraperitoneally at a dose of 100 μg/mouse every 3 days (*n* = 5 mice per group). Data are displayed as mean ± SEM. Significance in **D** was determined by 2-tailed *t* test and by 2-way ANOVA in **E**. Bonferroni’s correction was applied for multiple comparisons in **D** and **E**.

In tumors from mice treated with YJ1206, protein levels of phosphorylated RPA2 (p-RPA2-S33) and γH2AX, markers associated with TRC (39, 40), were markedly elevated (Supplemental Figure 8, C and D), supporting the conclusion that YJ1206 also induces TRC formation in vivo. We next examined whether tumors from YJ1206-treated animals showed increased levels of STING signaling. RNA sequencing revealed that STING activity signatures, as well as signatures that could be induced by STING activation (8), were significantly enriched in tumors from mice treated with YJ1206 compared with vehicle (Figure 5, A and B). Histological staining further revealed that administration of YJ1206 significantly elevated the levels of p-STING and p-IRF3 in tumor samples (Figure 5, C and D), demonstrating that YJ1206 treatment induced STING activation in vivo. STING activation is known to promote MHC-I expression (9), and as expected, tumor-specific MHC-I expression was significantly enhanced following YJ1206 treatment (Figure 5, C and D).

We also knocked out *Sting1* (the gene encoding STING) in tumor cells (Supplemental Figure 4A and Supplemental Figure 8E) and found that loss of *Sting1* partially rescued tumor growth under the combinatorial treatment of YJ1206 and anti–PD-1 (Supplemental Figure 8F and Figure 5E. To evaluate the role of host-derived STING, we performed the same combination treatment in *Sting1*-KO mice. In this context, tumors with *Sting1* deletion were resistant to low-dose YJ1206 combined with anti–PD-1 (Figure 5E). These findings collectively highlight that both tumor-intrinsic and host STING are essential for the full therapeutic efficacy of combined YJ1206 and anti–PD-1 treatment. This supports the concept that tumor-derived DNA can be transferred into the cytosol of antigen-presenting cells, thereby activating STING signaling in antigen-presenting cells and promoting CD8^+^ T cell–mediated antitumor immunity (41–44).

**Figure 6:**
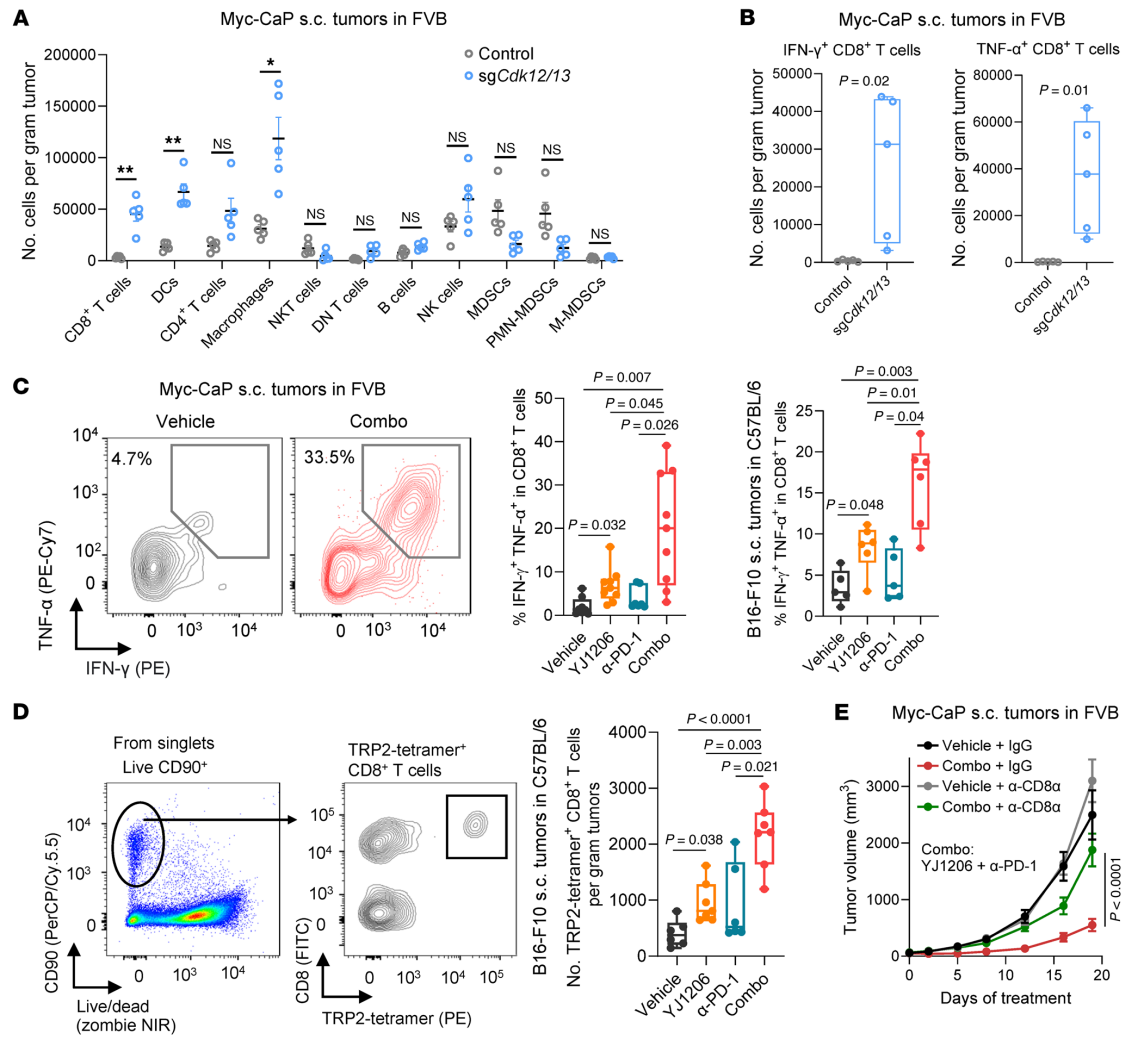
Antitumor activity of CDK12/13 degradation is CD8^+^ T cell dependent. (**A**) Quantification of flow cytometry showing the absolute number of the indicated immune cell populations in subcutaneous (s.c.) tumors derived from Myc-CaP cells with or without *Cdk12/13* depletion (sg*Cdk12/13*). DCs, dendritic cells; PMN-MDSCs, polymorphonuclear myeloid-derived suppressor cells; M-MDSCs, monocytic myeloid-derived suppressor cells. (**B**) Quantification of flow cytometry showing the absolute number of IFN-γ^+^ or TNF-α^+^ CD8^+^ T cells in tumors from **A**. (**C**) Representative images (left) or quantification (right) of flow cytometry measuring the proportion of IFN-γ^+^ and TNF-α^+^ CD8^+^ T cells in the indicated tumor models treated with vehicle, α-PD-1, YJ1206, or the combination (combo) of α-PD-1 and YJ1206 (*n* = 5–9 mice per group). (**D**) Left: Gating strategy for CD8^+^ T cells specific to TRP2 in flow cytometry. Right: Quantification of flow cytometry measuring the absolute number of CD8^+^ T cells specific to TRP2 in the indicated tumor models treated as in **C** (*n* = 6–7 mice per group). (**E**) Growth curves of s.c. tumors derived from Myc-CaP cells in FVB mice treated with the combination (combo) of α-PD-1 and YJ1206, following CD8^+^ T cell depletion by anti-CD8 antibody treatment (α-CD8a). Mice without CD8^+^ T cell depletion (IgG treated) were used as control (*n* = 5–10 mice per group). YJ1206 (100 mg/kg) was given orally 3 times per week, and α-PD-1 (200 μg/mouse) was administered intraperitoneally every 3 days. Data in **A** and **E** are displayed as mean ± SEM. Data in **B**–**D** are presented as box-and-whisker plots, with the median (center line), 25th–75th percentiles (box), and minimum to maximum values (whiskers). Significance in **A**–**D** was determined by 2-tailed *t* test and by 2-way ANOVA in **E**. NS, not significant. Bonferroni’s correction was applied for multiple comparisons.

In agreement with this, immune profiling (Supplemental Figure 9, A–E) revealed that CD8^+^ T cells and dendritic cells were among the most significantly increased populations of cells in tumors with *Cdk12/13* depletion compared with control (Figure 6A). Moreover, activated CD8^+^ T cells were also significantly increased (Figure 6B). Consistently, a significant increase in activated CD8^+^ T cells was also observed in tumors from YJ1206-treated mice (Figure 6C). Furthermore, while anti–PD-1 monotherapy only mildly elevated the proportion of active CD8^+^ T cells, the combination of anti–PD-1 and YJ1206 led to the most pronounced increase in intratumoral activated CD8^+^ T cells (Figure 6C). Importantly, the absolute number and fraction of tumor antigen–specific CD8^+^ T cells were significantly increased in tumors from mice treated with YJ1206 alone or in combination with anti–PD-1 (Figure 6D and Supplemental Figure 9F). To further assess the functional importance of CD8^+^ T cells, we treated mice with an anti-CD8 antibody, which significantly reduced the therapeutic efficacy of the YJ1206 and anti–PD-1 combination (Figure 6E). Collectively, these findings underscore the essential role of CD8^+^ T cells in mediating the antitumor effects of YJ1206 and anti–PD-1 combination therapy.

## Discussion

**Figure 7:**
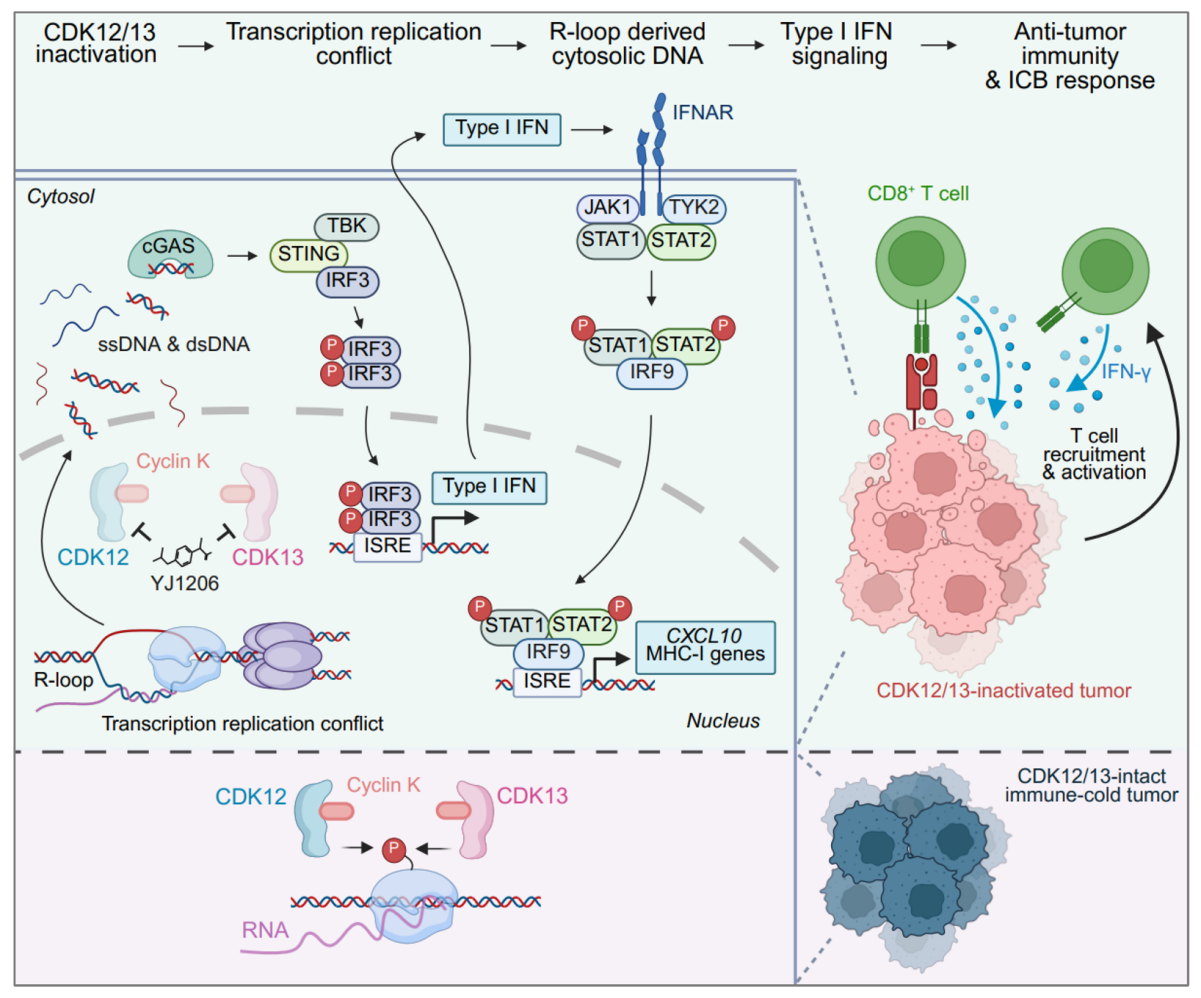
Mechanistic model of CDK12/13 antagonism enhancing antitumor immunity. Schematic illustrating that inactivation of CDK12/13 promotes formation of TRCs and R-loops, leading to an increase in cytosolic DNA. This, in turn, activates the cGAS/STING pathway, which enhances cancer cell killing mediated by CD8^+^ T cells.

This study is the first to our knowledge to establish an association between diminished expression of *CDK12/13* and heightened STING activity, identifying *CDK12/13* expression as a predictive biomarker for ICB response and patient survival in ICB-treated cohorts in a pan-cancer context. CDK12/13, by complexing with cyclin K, play vital roles in the phosphorylation of RNAPII, ensuring proper transcriptional elongation and processivity (Figure 7) (13–16). We demonstrate for the first time to our knowledge that simultaneous depletion of *Cdk12* and *Cdk13* is crucial to achieve the most robust TRC formation and R-loop accumulation, whereas depletion of either *Cdk12* or *Cdk13* alone results in only moderate effects. Consequently, dual depletion of *Cdk12/13* leads to the strongest activation of STING signaling and the most pronounced enhancement of ICB response. Notably, targeted degradation of CDK12/13 with YJ1206 treatment was evaluated in combination with ICB in a broad range of cancer types, including prostate cancer, melanoma, colon cancer, and lung cancer, where it significantly delays tumor growth and enhances ICB efficacy. Importantly, this effect is dependent on both STING activation and CD8^+^ T cells (Figure 7). Collectively, our data position CDK12/13 as predictive biomarkers for clinical outcomes in ICB-treated cohorts and establish pharmacologic antagonism of these kinases as a promising strategy to enhance ICB response, addressing a critical unmet clinical need. Our findings underscore the urgency of initiating clinical trials to evaluate the combination of CDK12/13 antagonism and ICB therapy as an approach to improve cancer treatment outcomes.

The mechanisms linking CDK12 inactivation to increased intratumoral T cell presence in advanced prostate cancer (18) and the development of preneoplastic lesions with lymphocytic infiltration in the murine prostate epithelium (14) have remained unclear. Here, we provide the first evidence to our knowledge that CDK12 inactivation activates the cGAS/STING pathway, which in turn drives T cell recruitment in CDK12-deficient cancers. Furthermore, we demonstrate that CDK12/13 antagonism, through either degradation or inhibition, effectively activates the STING pathway, sensitizing resistant tumors to ICB. These findings support CDK12/13 antagonism as a promising strategy to enhance STING activation and improve the efficacy of cancer immunotherapies. Recognizing the importance of STING activation in eliciting antitumor responses and enhancing ICB efficacy (7–9, 21), numerous clinical trials have been launched to investigate the potential of STING agonists in treating various cancer types or improving response to ICB. While many STING agonists have demonstrated promising efficacy in preclinical studies, their translation into the clinic has faced substantial challenges (45–47). One of the primary barriers is their suboptimal pharmacokinetic properties, which often necessitate direct intratumoral administration to achieve therapeutic concentrations at the tumor site (45, 46). Moreover, their poor pharmacokinetics result in a lack of oral bioavailability, limiting their widespread clinical application, as systemic administration is generally preferred for practical treatment regimens. Additionally, most STING agonists, due to their inherent negative charge, cannot effectively cross the plasma membrane of cells, which itself carries a negative charge (48). This charge-based limitation impedes their cellular uptake, reducing their efficacy in initiating STING-mediated immune responses. In this study, we demonstrate that the orally bioavailable PROTAC degrader of CDK12/13, YJ1206, activates the STING signaling pathway in a TRC-dependent manner. With its promising pharmacokinetic and pharmacodynamic properties, along with a favorable safety profile (20), YJ1206 emerges as a compelling candidate for clinical evaluation, particularly for its oral bioavailability and potential to enhance ICB efficacy by activating STING signaling. Notably, a molecular glue degrader of cyclin K, CT7439, which inhibits CDK12/13, has progressed to a phase I clinical trial (ClinicalTrials.gov NCT06600789) for patients with advanced solid tumors. Additionally, several other companies are developing CDK12/13 inhibitors, underscoring the substantial therapeutic potential of targeting these kinases for cancer treatment.

Although our study demonstrates that inactivation of CDK12/13 markedly activates the STING signaling pathway and enhances efficacy of ICB, these observations were obtained in preclinical models. While the results are promising, further investigation in clinical settings is necessary to fully evaluate the potential of CDK12/13 degraders or inhibitors as a strategy to improve ICB responses. The continued development of these compounds could offer an approach to activate STING signaling in tumors and boost ICB efficacy. Future clinical trials will be crucial to confirm the translational potential of these findings and determine the safety, pharmacokinetics, and therapeutic benefits of targeting CDK12/13 in conjunction with ICB.

## Methods

### Sex as a biological variable.

For drug efficacy studies, Myc-CaP (murine prostate cancer) tumors were established in male FVB mice, while B16-F10 (melanoma), LLC (lung cancer), and CT26 (colon cancer) tumors were established in female mice. Treatment with YJ1206, either alone or in combination with anti–PD-1, demonstrated comparable efficacy across these models. These findings suggest that the sex of the animals may not significantly influence treatment efficacy. Clinical sequencing data included in this study were collected from both male and female patients through the MI-ONCOSEQ program (see *Human studies* below).

### Animal experiments.

Mice were housed at a maximum density of 5 per cage, provided with regular chow, nesting material, and igloos, and maintained on a 12-hour light-dark cycle with controlled humidity (30%–70%) and temperature (20°C–26.1°C). BALB/c (strain 028), FVB (strain 207), and C57BL/6 (strain 027) mice were obtained from Charles River. NOD.Cg-*Prkdc*^scid^ *Il2rg*^tm1Wjl^/SzJ (NSG; strain 005557) mice and the *Sting1*-KO mice (BALB/c-*Sting1*^em3Vnce^/J; strain 036638) were acquired from the Jackson Laboratory. Mice were used to establish subcutaneous tumors. Tumor models were created by subcutaneously injecting 3 million Myc-CaP cells into male FVB mice, 0.4 million B16-F10 or LLC cells into female C57BL/6 mice, and 0.5 million CT26 cells into BALB/c female mice at each site. Treatments were initiated once tumors reached 50–100 mm^3^ in size.

The vehicle solution consisted of 20% PEG400, 6% Cremophor EL, and 74% PBS. YJ1206 or vehicle was administered orally at a dose of 100 mg/kg, 3 times per week. Anti–PD-1 or IgG was administered intraperitoneally at a dose of 200 μg/mouse every 3 days. Following the treatment regimen, mice were sacrificed, and organs and tumors were collected for further analysis. CD8^+^ T cell depletion was performed as described previously (49). Anti–mouse CD8α (clone 2.43) or its corresponding isotype control were purchased from BioXcell. The antibody was administered intraperitoneally 1 day before tumor cell inoculation, at a loading dose of 400 μg per mouse, followed by 100 μg per mouse every 3 days until the conclusion of the experiment. Intratumoral injections of cGAMP (InvivoGen, TLRL-NACGA23-02) were administered at a dose of 2.5 μg in 25 μL, 3 times every other day.

### Immunoblot analysis.

Following treatment under varying conditions, cell lysates were prepared in RIPA buffer (Thermo Fisher Scientific), supplemented with cOmplete protease inhibitor cocktail (Roche, 4693159001) and PhosSTOP phosphatase inhibitor (Roche, 4906837001). Protein concentrations were determined using the Pierce BCA Protein Assay Kit (Bio-Rad) following the manufacturer’s instructions. Equal amounts of protein from each sample were loaded onto NuPAGE 3%–8% Tris-Acetate or 4%–12% Bis-Tris protein gels (Thermo Fisher Scientific) and transferred to membranes. Membranes were blocked with 5% nonfat milk at room temperature for 1 hour, followed by overnight incubation at 4°C with primary antibodies, which were diluted using 5% nonfat milk or 5% BSA (Sigma-Aldrich, A9647) in PBST (0.1% Tween 20 in PBS). Detection was performed with HRP-conjugated secondary antibodies, and images were acquired using an Odyssey Fc Imager (LI-COR Biosciences). Antibodies used in this study included anti-CDK12 (Proteintech, 26816-1-AP), anti-CDK13 (Proteintech, 30461-1-AP), anti–p-IRF3 (Cell Signaling Technology, 29047S), anti–p-TBK1 (Cell Signaling Technology, 5483S), anti-cGAS (Cell Signaling Technology, 31659S), anti-p65 (Cell Signaling Technology, 8242T), anti-STING (Cell Signaling Technology, 13647S), anti-GAPDH (Cell Signaling Technology, 3683S), anti-IRF3 (Cell Signaling Technology, 4302S), anti–p-Ser2 (Cell Signaling Technology, 13499), anti-vinculin (Cell Signaling Technology, 18799S), anti–mouse p-STING (Cell Signaling Technology, 72971S), anti–human p-STING (Cell Signaling Technology, 19781S), anti-TBK1 (Cell Signaling Technology, 3013S), anti-γH2AX (Abcam, ab11174), and anti–IFN-αR1 (Santa Cruz Biotechnology, sc-53591). Secondary antibodies included Rabbit IgG HRP-linked whole Ab (Cytiva, NA934-1ML) and Mouse IgG HRP Linked Whole Ab (Cytiva, NA931-1ML).

### Transfection.

Cells were seeded in 6-well plates at approximately 30%–40% confluence and incubated overnight at 37°C with 5% CO_2_. RNAiMAX (3.75 μL; Thermo Fisher Scientific, 13778075) was added to 125 μL of Opti-MEM (Thermo Fisher Scientific, 31985062) and combined with an additional 125 μL of Opti-MEM containing 500 nM si*Cdk12* and/or si*Cdk13* (si*Cdk12*, Horizon Discovery, J-064510-06-0050; si*Cdk13*, Horizon Discovery, J-045210-05-0050). The mixture was incubated at room temperature for 5 minutes before being added to the cells.

### Cell lines.

Cell lines were obtained as reported elsewhere (49, 50). The B16-F10, Myc-CaP, CT26, LLC, 22RV1, VCaP, and PC3 cell lines were purchased from the American Type Culture Collection (ATCC). Manufacturer’s guidelines were followed to culture all cell lines. Cell line pellets were regularly harvested and submitted to the LabCorp Cell Line Testing division to confirm authentication. To ensure the absence of contamination, all cell lines were tested for mycoplasma every 2 weeks.

Stable cell lines were generated as previously described (49, 51, 52). Briefly, single guide RNAs (sgRNAs) targeting early exons of *Cdk12* or *Cdk13* were evaluated for off-target effects using Off-Spotter (https://cm.jefferson.edu/Off-Spotter/). The sgRNAs with minimal off-target potential were chosen (Supplemental Table 3 lists the sgRNAs used in this study). The Golden Gate assembly method was employed to construct the sgRNAs to lentiCRISPR v2 vector (Addgene, 52961). Sanger sequencing confirmed successful construction. The resulting vectors were transfected into target Myc-CaP cells using Lipofectamine 3000 (Thermo Fisher Scientific, L3000001). One day after transfection, puromycin was used at 10 μg/mL to select for transfected cells. A SONY SH800S cell sorter was used to sort single cells to 96-well plates. Colonies from single cells were expanded, and gene depletion was evaluated by Sanger sequencing and immunoblotting. All generated cell lines were routinely tested to ensure they remained free of mycoplasma contamination.

### Reverse transcription–quantitative PCR.

Total RNA was extracted using the RNeasy Mini Kit (Qiagen, 74104) following the manufacturer’s instructions. RNA concentration was measured using a NanoDrop spectrophotometer. For cDNA synthesis, 1000 ng of total RNA was used with the Maxima First Strand cDNA Synthesis Kit for reverse transcription–quantitative PCR (RT-qPCR) (Thermo Fisher Scientific, 12574026). qPCR was performed in triplicate using SYBR Green reagents on a QuantStudio 6 Real-Time PCR System (Applied Biosystems). mRNA expression levels were quantified using the ΔCt method and normalized to *ACTB* expression. Primers were synthesized by Integrated DNA Technologies, with sequences provided in Supplemental Table 4.

### ELISA.

Cells were treated with YJ1206 for 24 hours or with si*CDK12* with and without si*CDK13* for 36 hours. Supernatants were collected and centrifuged at 500*g* for 3 minutes for IFN-β ELISA analysis. Cells were lysed using M-PER Mammalian Protein Extraction Reagent (Thermo Fisher Scientific, 78501) for cGAMP ELISA. Protein concentrations were measured with the Pierce BCA Protein Assay Kit (Thermo Fisher Scientific) according to the manufacturer’s instructions. IFN-β ELISA (R&D Systems, DY8234-05 and DY008B) and cGAMP ELISA (Cayman Chemical, 501700) kits were used per the manufacturer’s protocols.

### RNA sequencing.

Myc-CaP cells were exposed to 1 μM of the CDK12/13 degrader YJ1206 for 24 hours. Total RNA was then isolated using the RNeasy Mini Kit (Qiagen) and quantified using the NanoDrop 2000 spectrophotometer (Thermo Fisher Scientific). For RNA sequencing, libraries were constructed with the KAPA RNA HyperPrep Kit with RiboErase (HMR) (Roche, 08098140702). Briefly, 800 ng of total RNA underwent ribosomal RNA depletion via enzymatic digestion, followed by first- and second-strand cDNA synthesis, end repair, A-tailing, and adapter ligation using NEB adapters. Fragments ranging from 250 to 300 bp were size-selected using a 2-step AMPure XP bead purification, and libraries were amplified by PCR with KAPA HiFi HotStart ReadyMix and NEB dual-index primers (Roche, E6440L). Final libraries were assessed for quality and fragment distribution using the Agilent 2100 Bioanalyzer. Sequencing was performed on the Illumina NovaSeq 6000 platform to generate 150-bp paired-end reads, targeting a depth of 20–30 million read pairs per sample. Downstream data processing and analysis followed previously described protocols (49). GSEA was conducted using the STING pathway activation signature reported by Wu et al. (8).

### Human studies.

The MI-ONCOSEQ clinical sequencing program at the Michigan Center for Translational Pathology (MCTP) sequenced samples from patients recruited at the University of Michigan hospital (17, 53–55). Data from samples collected prior to ICB treatment were used in the study. Treatment response was evaluated based on the RECIST1.1 criteria, excluding cases of pseudoprogression based on imRECIST criteria (56). Sequencing of patient samples was conducted by the MCTP Clinical Laboratory Improvement Amendments (CLIA)–certified laboratory, adhering to approved protocols and ethical guidelines as previously described (17, 54, 57). All patients provided written informed consent. Demographic data, including race and ethnicity, were collected in alignment with NIH guidelines. Classifications were based on self-reported data by the participants using categories defined by the investigators to ensure consistency with federal reporting requirements. Supplemental Table 1 details the full clinical and demographic characteristics of the cohort.

### Analysis of public gene expression data.

Public gene expression data were acquired from the KM plotter (24, 25) (https://kmplot.com/analysis/), cBioPortal (58–60) (https://www.cbioportal.org/), or Tumor Immunotherapy Gene Expression Resource (http://tiger.canceromics.org/#/). Two metastatic melanoma datasets, Van Allen et al. (22) and Riaz et al. (23), were used in this study. Data from samples collected before treatment were utilized to assess whether expression of *CDK12/13* could predict treatment response and patient survival. In the dataset from Riaz et al. (23), data from samples in M1b and M1c stages were utilized. Data from the KM plotter were obtained from pretreatment samples of patients who received ICB therapy. For CDK12/13, we used the average *z*-scored expression of *CDK12* and *CDK13* genes, while the STING activity signature consisted of *CCL5*, *IFNB1*, *CXCL10*, *ISG15*, *ISG20*, and *IRF7* (61–63).

### Spatial transcriptomics.

Spatial transcriptomics was performed using the Visium HD, Human Transcriptome (10× Genomics, 1000673) and Visium CytAssist (10× Genomics, 1000441) following the instructions from the manufacturer. Briefly, 5-μm FFPE tissue on tissue slides was stained with H&E, imaged, destained, and decrosslinked. Tissue was next permeabilized and hybridized with human transcriptomic probes overnight. Hybridized probes were then transferred to the Visium slide, which contains millions of barcodes, through the CytAssist instrument. Next, cDNAs were synthesized and amplified with sample indexes for the final libraries. The quality of the libraries was evaluated by the Agilent Bioanalyzer. For analyzing the spatial transcriptomics data, FASTQ files were generated from the raw base call files using 10× Genomics Space Ranger v3.1.0 mkfastq. The gene count matrices at an 8-μm bin size were generated from the FASTQ files using the count command and the 10× Genomics–supplied human probe-set reference. The data were processed using Seurat v5.1.0 (64). The 8-μm bin spots were clustered using sketch-based clustering and integrated across conditions for downstream analyses according to the Seurat Visium HD workflow. Each spot was annotated using SingleR (65) and an in-house reference built from public single-cell RNA sequencing datasets (66, 67). Pseudobulk matrices were generated for the annotated cell types and used for gene-set enrichment comparison across conditions. Genes were ranked based on log_2_(fold change) and pathway analysis was conducted using clusterProfiler (68). Pathways of interest were taken from the Molecular Signatures Database (MSigDB) Hallmark gene set collection (69) and gene ontology (GO) Biological Processes collection (70, 71). UCell (72) was used to calculate per-spot gene-set enrichment scores. The STING activity signature applied in the GSEA with the spatial transcriptomics data is derived from the publication by Wu et al. (8).

### Analysis of single-cell RNA sequencing in ICB-treated cohorts.

We previously compiled a curated list of single-cell RNA-sequencing datasets encompassing pan-cancer ICB-treated cohorts (73). These include skin cancers such as melanoma (74–77) and basal cell carcinoma (78). Additionally, investigations have extended to breast cancer subtypes (79) like triple-negative, HER2-positive, and estrogen receptor (ER)–positive, as well as kidney cancer, specifically clear cell renal carcinoma (80), and liver cancers (81) such as hepatocellular carcinoma and intrahepatic cholangiocarcinoma. In this resource, we have grouped partial responders, responders, complete responders, and expanded (tumor samples with T cell expansion after ICB treatment) as “Favorable” outcomes, whereas nonresponders, stable disease, and non-expanded are classified as “Unfavorable” outcomes. This resource was employed to assess CDK12/13 and STING activity in single cells. Each dataset was subsetted for tumor cells. Single-cell analysis was performed using the Seurat package (v5.1.0). Data normalization was performed using log1p normalization, and clustering was undertaken using Seurat’s unsupervised graph-based clustering approach. To evaluate CDK12/13 and STING activity expression per patient, the Average Expression function from Seurat was employed to extract the patient-specific cluster expression. For CDK12/13, we utilized the average expression of *CDK12* and *CDK13* genes. For STING activity, we used *CCL5*, *CXCL10*, *ISG15*, *ISG20*, and *IRF7* gene signature.

### Flow cytometry.

Cells were treated with 1 μM YJ1206 for 24 hours or with si*Cdk12* with and without si*Cdk13* for 36 hours. Following treatment, cells were washed once with PBS, trypsinized, and neutralized with MACS buffer (2% FBS, 2 mM EDTA in PBS). The cell suspension was then filtered into FACS tubes (Thermo Fisher Scientific, 08-771-23), centrifuged at 500*g* for 5 minutes, and the supernatant was carefully aspirated. Cells were resuspended in 100 μL PBS mixed with Zombie NIR viability dye (BioLegend, 423106) and stained at room temperature for 5 minutes in the dark. Next, MHC-I antibodies — anti–H-2Kq (BD Biosciences, 742296) and anti–H-2Dq/H-2Lq (BD Biosciences, 744853) for Myc-CaP cells; anti–H-2Kb (BD Biosciences, 553570) and anti–H-2Db (Thermo Fisher Scientific, 12-5999-83) for B16-F10 cells; anti–H-2Kd (BD Biosciences, 562004) and anti–H-2Dd (BD Biosciences, 553580) for CT26 cells — and anti–PD-L1 antibody (BD Pharmingen, 568303), diluted in 50 μL MACS buffer, were added and incubated with the cells at room temperature for 12 minutes in the dark. Cells were then washed with 1 mL MACS buffer, centrifuged, and the supernatant was aspirated. The cell pellet was resuspended in 55 μL fixative buffer and incubated at room temperature for 12 minutes, followed by the addition of 200 μL MACS buffer. The samples were analyzed on a SH800S cell sorter (Sony Biotechnology), with data collected from over 10,000 cells. FlowJo software (version 10.8.2) was used for data analysis.

For immune profiling, tissues were first weighed and minced. These tissue pieces were then digested with 0.5 mg/mL collagenase D (Roche, COLLD-RO) and 0.25 mg/mL DNase I (Roche, 10104159001) in PBS containing 2% FBS, at 37°C for 30 minutes. After digestion, the tissue suspensions were applied to cell strainers (70 μm), and the filtrates were layered onto density gradient media (Histopaque-1119 and Histopaque-1077; Sigma-Aldrich, 11191-100ML and 10771-100ML, respectively) in centrifuge tubes. Following centrifugation, the cell layer was collected and washed with MACS buffer. For staining of T cell intracellular markers, the isolated cells were then incubated in RPMI 1640 (Gibco, 11875093) supplemented with 10% FBS, 50 U/mL penicillin-streptomycin, 10 mM HEPES, 27.5 μM β-mercaptoethanol, 200 ng/mL phorbol 12-myristate 13-acetate (PMA), 1000 ng/mL ionomycin, 1× brefeldin A, and 1× monensin. Incubation was conducted at 37°C for 4 hours (49). After incubation, cells were stained with Zombie Green (BioLegend, 423112) and blocked with anti–mouse CD16/CD32 (BioLegend, 156604), following the user manuals. Surface markers were stained in MACS buffer at room temperature for 12 minutes. The cells were then washed with MACS buffer. For intracellular staining, cells were fixed and permeabilized using the Foxp3/Transcription Factor Staining Buffer Set (Thermo Fisher Scientific, 00-5523-00), following the manufacturer’s instructions. Intracellular markers were stained for 12 minutes at room temperature, followed by a wash with 2 mL of 1× Permeabilization Buffer. Flow cytometry analysis was carried out on a BD LSRFortessa Cell Analyzer, with Absolute Counting Beads (Thermo Fisher Scientific, C36950) used for quantification. The surface antibodies used in this study included anti-CD45 (BD Biosciences, 550994), anti-CD3 (BioLegend, 100237), anti-CD90.1 (BD Biosciences, 563770), anti-CD90.2 (BioLegend, 140327), anti-CD8 (BioLegend, 100742), anti-CD4 (BD Biosciences, 553051), anti-CD11c (Thermo Fisher Scientific, 25-0114-82), anti-CD11b (BioLegend, 101208), anti–Gr-1 (BioLegend, 108422), anti–MHC-II (BD Biosciences, 562564), anti-F4/80 (BD Biosciences, 565613), anti–Ly-6C (BioLegend, 128033), anti–Ly-6G (BioLegend, 127624), anti-CD19 (BioLegend, 115541), and anti–NK-1.1 (BioLegend, 108714). Intracellular markers were detected using anti-Ki67 (Thermo Fisher Scientific, 56-5698-82), anti–granzyme B (BioLegend, 372208), and anti–IFN-γ (BD Biosciences, 562333).

For TRP2-tetramer staining, tumor tissues were processed, and cell suspensions were obtained as described above. The suspended cells were then incubated with Zombie NIR (BioLegend, 423106) and H-2K^b^ TRP2 Tetramer/PE (SVYDFFVWL; MBL International, TS-5004-1C) for 15 minutes. Following viability and tetramer staining, Fc receptors were blocked using anti–mouse CD16/CD32 antibody (BioLegend, 156604) for 2 minutes at room temperature. Cells were then incubated with anti-CD90.2 (BioLegend, 105338) and anti-CD8 (Thermo Fisher Scientific, MA5-16759) antibodies for 10 minutes. Samples were next fixed in PBS containing 0.5% paraformaldehyde, on ice for 1 hour. To enable quantification, Absolute Counting Beads (Thermo Fisher Scientific, C36950) were added according to the manufacturer’s instructions. Finally, samples were analyzed using a BD LSRFortessa flow cytometer.

### PLA.

Cells were seeded in 8-well chamber slides (Thermo Fisher Scientific, 154941PK) and treated with YJ1206 for 4 hours, or with si*Cdk12* with and without si*Cdk13* for 36 hours, with or without DRB (75 μM; Sigma-Aldrich, D1916) or triptolide (1 μM; Sigma-Aldrich, T3652) for 200 minutes. Prior to collection, cells were synchronized using 2 mM thymidine (Selleck Chemicals, S4803) for 12 hours. Cells were then washed with PBS, fixed with precooled methanol for 10 minutes, and permeabilized with 0.5% Triton X-100 in PBS for 10 minutes at room temperature. The Naveni TriFlex Cell MR kit (Navinci/Sapphire, TF.MR.100) was used following the manufacturer’s protocol. Briefly, primary antibodies against PCNA (1:500; Abcam, ab18197) and RNAPII RPB1 (1:500; BioLegend, 920204), were applied, followed by the secondary Navenibody R TF (1:40). Final images were acquired using a confocal microscope.

### Immunohistochemistry.

After embedding in paraffin, tumor tissues were sectioned, deparaffinized, and rehydrated. Antigen retrieval was performed using a buffer containing 10 mM sodium citrate and 0.05% Tween 20 (pH 6.0). The sections were then treated with 3% hydrogen peroxide to block endogenous peroxidase activity, followed by incubation with 5% goat serum diluted in PBS for blocking. Afterward, the sections were incubated overnight at 4°C with the primary antibody, then washed with PBS containing 0.1% Tween 20. The samples were subsequently incubated with a secondary antibody for 1 hour, washed again with PBS containing 0.1% Tween 20, and developed using 3,3′-diaminobenzidine (Vector Laboratories, SK-4100). Counterstaining was done by incubating the sections with hematoxylin for 2 minutes. After mounting, images of the stained sections were captured using a microscope. Quantification was carried out by deconvoluting the brown-stained areas in the images using the Fiji ImageJ software (https://imagej.net/software/fiji/downloads). The primary antibodies used in this procedure included p-IRF3 (Invitrogen, PA5-36775) and HRP-conjugated goat anti-rabbit IgG (Vector Laboratories, MP-7451-15).

### Immunofluorescence.

Myc-CaP and B16-F10 cells were seeded in 8-well chamber slides (Thermo Fisher Scientific, 154941PK) and treated with YJ1206 for 4 hours or with si*CDK12* with and without si*CDK13* for 36 hours, with or without DRB (75 μM; Sigma-Aldrich, D1916) for 200 minutes. Cells were washed twice with PBS, fixed in 4% paraformaldehyde (Santa Cruz Biotechnology, sc-281692), and permeabilized with 0.5% Triton X-100 at room temperature for 10 minutes. For RNase H treatment, cells were incubated with 120 U RNase H (Takara Bio, 2150A) in RNase H buffer (40 mmol/L Tris-HCl pH 8.0, 4 mmol/L MgCl_2_, 1 mmol/L dithiothreitol, 4% glycerol, and 0.003% BSA) for 4 hours at 37°C. Cells were then washed 3 times with PBS and blocked with MAXblock Blocking Medium (Active Motif, 15252) for 1 hour at 37°C. Primary antibodies γH2AX (1:1000; Abcam, ab11174) or DNA/RNA hybrid (1:50; EMD Millipore, MABE1095), diluted in PBS with 1% BSA and 0.3% Triton X-100, were added and incubated overnight at 4°C. After three 5-minute washes with PBS, cells were incubated with secondary antibodies at room temperature for 1 hour, washed again with PBS, and counterstained with DAPI. Slides were mounted with Prolong Gold Antifade Reagent (Thermo Fisher Scientific, P36935) and imaged on a confocal microscope.

Paraffin-embedded tumor sections were processed as described above. Following blocking with 10% goat serum in PBS, the sections were incubated overnight at 4°C with primary antibodies against γH2AX (1:2000; Abcam, ab11174) or p-RPA2 (S33) (1:500; Abclonal, AP1479), diluted in PBS containing 1% BSA. Subsequent washing, secondary antibody incubation, DAPI staining, mounting, and imaging were performed as described above.

For cytosolic dsDNA and ssDNA detection, cells were permeabilized with 0.01% Triton X-100 and 0.1% Tween 20 in PBS. Primary antibodies, dsDNA (1:100; Millipore, MAB1293), and ssDNA (1:100; Millipore, MAB3868), as well as secondary antibodies, were diluted in PBS with 0.005% Triton X-100, 0.05% Tween 20, and 1% BSA.

### TUNEL staining.

TUNEL staining was performed on paraffin-embedded tumor sections using the In Situ Cell Death Detection Kit (Roche) according to the manufacturer’s instructions. In brief, sections were deparaffinized, rehydrated, and treated with proteinase K, followed by incubation with the TUNEL reaction mixture. After washing, nuclei were counterstained with DAPI. Images were acquired using a fluorescence microscope, and apoptotic cells were quantified using Fiji ImageJ.

### Assessment of drug synergism.

The assessment of in vivo drug synergy was conducted using combPDX (https://licaih.shinyapps.io/CombPDX/), following the provided tutorial (82). Combination indexes were calculated based on the Highest Single Agent (HSA) reference model. A combination index greater than zero was considered indicative of supra-additive (synergistic) effects (82).

### Statistics.

All data were derived from independent biological replicates. Statistical analyses were performed using Prism version 8 (GraphPad Software) and R (v4.4.0) for transcriptomic data. Quantitative results are presented as mean ± SD or mean ± SEM, as specified in the figure legends. Statistical significance was defined as a *P* value of less than 0.05. For experiments involving multiple comparisons, corrections for false discovery were applied — Bonferroni’s method for small-scale datasets and the Benjamini-Hochberg procedure for RNA sequencing. For GSEA, statistical significance was assessed using a permutation-based enrichment score, with the normalized enrichment score (NES) and adjusted *P* value reported.

### Study approval.

All animal studies were conducted in compliance with the guidelines of the University of Michigan Institutional Animal Care and Use Committee (IACUC) and received committee approval. The use of clinical data in this study was approved by the University of Michigan Institutional Review Board (IRB, HUM00046018 and HUM00067928), and all patients involved in the MI-ONCOSEQ program provided written informed consent.

### Data availability.

Data acquired from sequencing have been deposited in the NCBI Gene Expression Omnibus (GEO GSE283227 and GSE283088). Values for all data points in graphs are reported in the Supporting Data Values file. All other data are available in the main manuscript or the supplemental materials.

## Author contributions

Authorship order among co–first authors was determined based on the relative extent of their contributions to experimental design, data acquisition, and manuscript preparation. YB, YC, JCYT, IK, WZ, KD, and AMC developed the concept. YB, YC, and AMC wrote the manuscript. YB and YC were responsible for designing and conducting experiments and analyzing the data. JCYT, FY, RM, SM, RP, JEC, and JKG helped design and perform the experiments and assisted in analyzing the data. GC, YZ, MG, and MC contributed to the analysis of sequencing data. SJM helped write and edit the manuscript. DRR, YMW, XC, FS, and RW helped design and perform the RNA sequencing. AT assisted with statistical analysis. XW, YQ, and AMC oversaw the study.

## Supplementary Material

Supplemental data

Unedited blot and gel images

Supplemental table 1

Supplemental table 2

Supporting data values

## Version 1 07/23/2025

In-Press Preview

## Version 2 09/16/2025

Electronic publication
