# Supplementary material for: CDK12/13 inactivation triggers STING-mediated antitumor immunity in preclinical models
Source: J Clin Invest. 2025 Jul 22;135(18):e193745. doi: 10.1172/JCI193745 (PMC12435847; doi:10.1172/JCI193745)
Supplement: Supplemental data [file jci-135-193745-s093.pdf]

Supplemental information for

CDK12/13 inactivation triggers STING-mediated anti-tumor immunity in pre-clinical models

Yi Bao[1,2,#], Yu Chang[1,2,#], Jean Ching-Yi Tien[1,2], Gabriel Cruz[1,2], Fan Yang[1,2], Rahul Mannan[1,2], Somnath Mahapatra[1,2], Radha Paturu[1,2], Xuhong Cao[1,2], Fengyun Su[1,2], Rui Wang[1,2], Yuping Zhang[1,2], Mahnoor Gondal[1,2,3], Jae Eun Choi[1,2], Jonathan K. Gurkan[1], Stephanie J. Miner[1,2], Dan R. Robinson[1,2], Yi-Mi Wu[1,2], Licheng Zhou[4,5], Zhen Wang[4,5], Ilona Kryczek[6,7], Xiaoju Wang[1,2,8], Marcin Cieslik[1,2,3], Yuanyuan Qiao[1,2,8], Alexander Tsodikov[9], Weiping Zou[1,2,6,7,8], Ke Ding[4,*], and Arul M. Chinnaiyan[1,2,8,10,11,*]

[1] Michigan Center for Translational Pathology, University of Michigan, Ann Arbor, MI, 48109
[2] Department of Pathology, University of Michigan, Ann Arbor, MI, 48109
[3] Department of Computational Medicine and Bioinformatics, University of Michigan, Ann Arbor, MI, 48109
[4] State Key Laboratory of Chemical Biology, Shanghai Institute of Organic Chemistry, Chinese Academy of Sciences, Shanghai 200032, People's Republic of China
[5] International Cooperative Laboratory of Traditional Chinese Medicine Modernization and Innovative Drug Discovery of Chinese Ministry of Education (MOE), Guangzhou City Key Laboratory of Precision Chemical Drug Development, College of Pharmacy, Jinan University, Guangzhou 511400, People's Republic of China
[6] Department of Surgery, University of Michigan, Ann Arbor, MI, 48109
[7] Center of Excellence for Cancer Immunology and Immunotherapy, University of Michigan, Ann Arbor, MI, 48109
[8] Rogel Cancer Center, University of Michigan, Ann Arbor, MI, 48109
[9] Department of Biostatistics, University of Michigan, Ann Arbor, MI, 48109
[10] Howard Hughes Medical Institute, University of Michigan, Ann Arbor, MI, 48109
[11] Department of Urology, University of Michigan, Ann Arbor, MI, 48109
[#] These authors contributed equally

***Correspondence to:**
Arul M. Chinnaiyan, M.D., Ph.D.
University of Michigan
1500 E. Medical Center Dr.
5316 Rogel Cancer Center
Ann Arbor, MI 48109
Phone: (734) 615-4062
Email: arul@med.umich.edu

Ke Ding, Ph.D.
#345 Lingling Road, Shanghai 200032
Phone: (8621) 5492 5100
Email: dingk@sioc.ac.cn

Conflict of Interest Statement: A.M.C. is a co-founder and serves on the Scientific Advisory Board of Lynx Dx, Esanik Therapeutics, Medsyn, and Flamingo Therapeutics. A.M.C. is a scientific consultant for EdenRoc, Proteovant, Aurigene Oncology, RAPPTA, Belharra, and Tempus. K.D. provides advisory services to Kinoteck Therapeutics and has received financial

support from Livzon Pharmaceutical Group. W.Z. acts as a scientific advisor for NGM, CrownBio, Cstone, Proteovant, Hengenix, NextCure, and Intergalactic. Patents related to the CDK12/13 degrader, YJ1206, have been filed by both the University of Michigan and the Shanghai Institute of Organic Chemistry, with A.M.C., K.D., Y.C., X.W., and J.C.T. recognized as co-inventors.

This PDF file includes:

Supplementary Figures S1 to S9
Supplementary Tables S3 to S4

Other Supporting Information for this manuscript include the following:

Supplementary Tables S1 and S2 (Excel file)

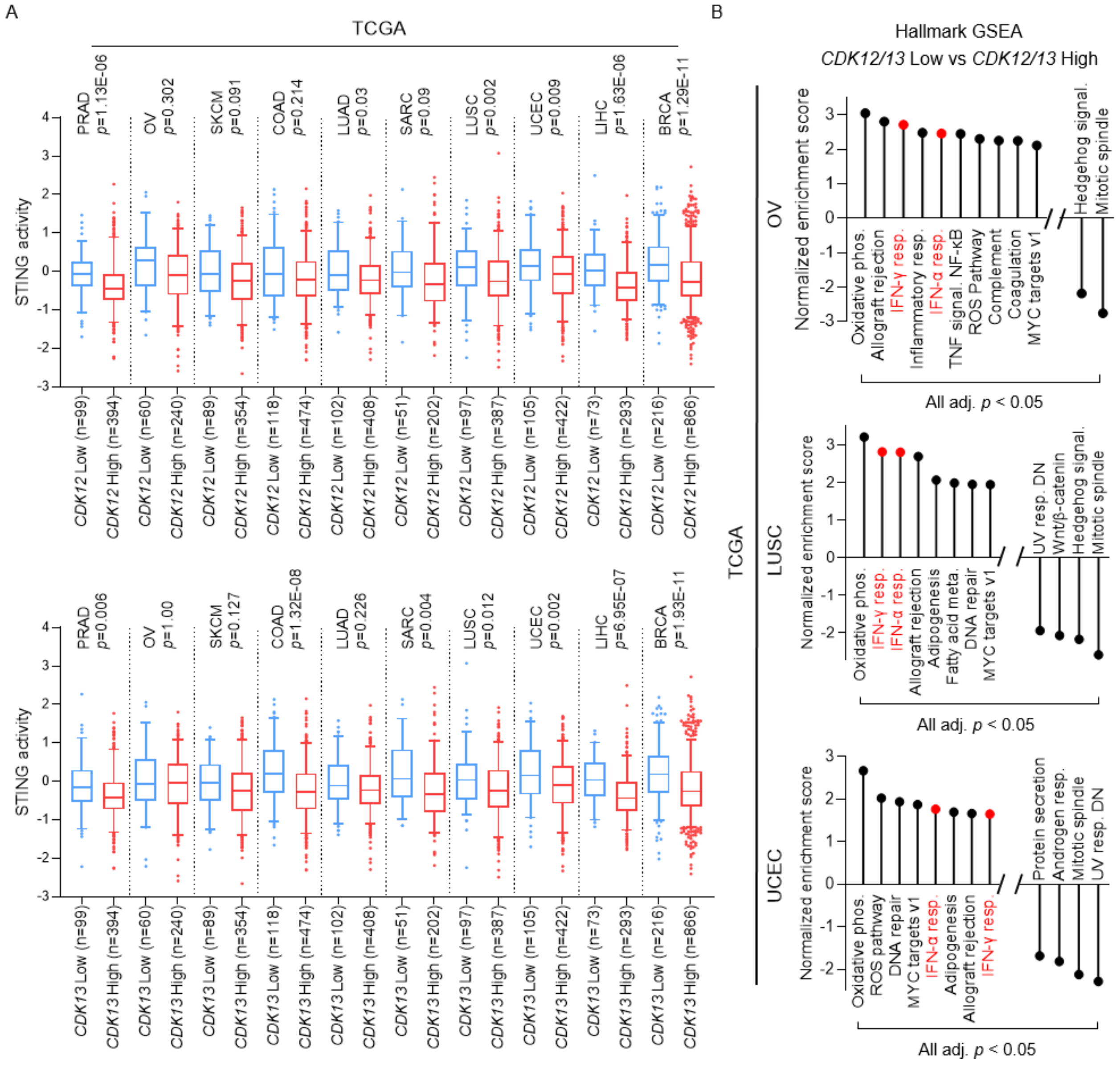

Supplemental Figure S1: **A**, Association between expression of *CDK12* (top) or *CDK13* (bottom) and STING activity in the indicated cancer types. Data were acquired from TCGA datasets. PRAD: prostate adenocarcinoma; OV: ovarian serous cystadenocarcinoma; SKCM: skin cutaneous melanoma; COAD: colon adenocarcinoma; LUAD: Lung adenocarcinoma; SARC: sarcoma; LUSC: lung squamous cell carcinoma; UCEC: uterine corpus endometrial carcinoma; LIHC: liver hepatocellular carcinoma; BRCA: breast invasive carcinoma. **B**, Top pathways enriched by GSEA,

utilizing the MSigDB Hallmark database, in *CDK12/13* low versus *CDK12/13* high group in the indicated cancers. Type-I and -II IFN responses are highlighted in red. Data were acquired from TCGA datasets.

Statistics in **A** were acquired by two-tailed t test with Bonferroni correction. Data in **A** are shown as box-and-whisker plots with the median (center line), 25th–75th percentiles (box), 10th–90th percentiles (whiskers), and outliers beyond the whiskers. Low expression of *CDK12*, *CDK13*, or *CDK12/13* was defined as the bottom 20th percentile within each cohort.

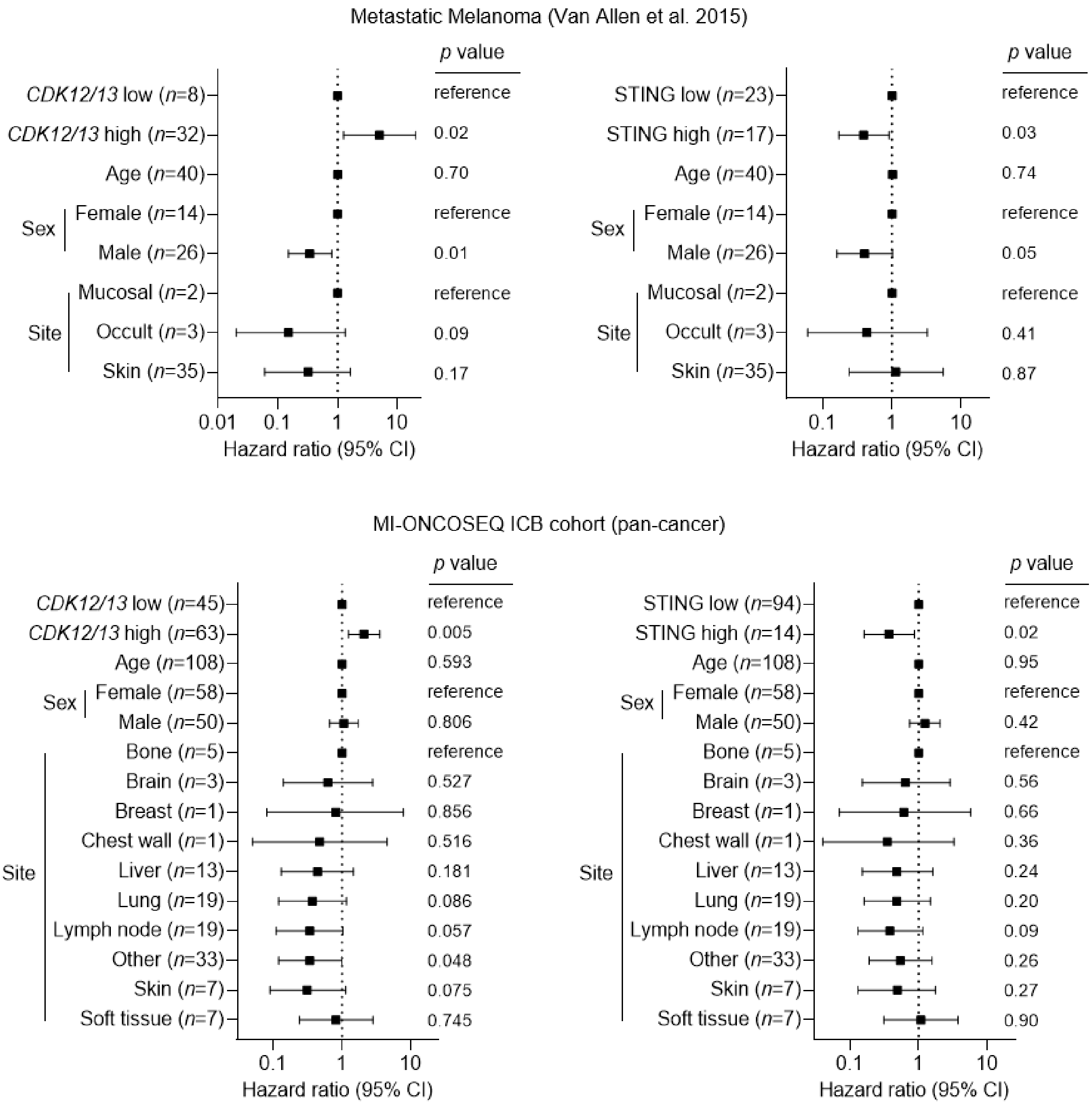

Supplemental Figure S2: Multiple linear regression analysis of overall survival was performed using the indicated variables, including pre-treatment *CDK12/13* expression (left) or pre-treatment STING activity signature expression (right), across the specified ICB cohorts. CI: confidence interval.

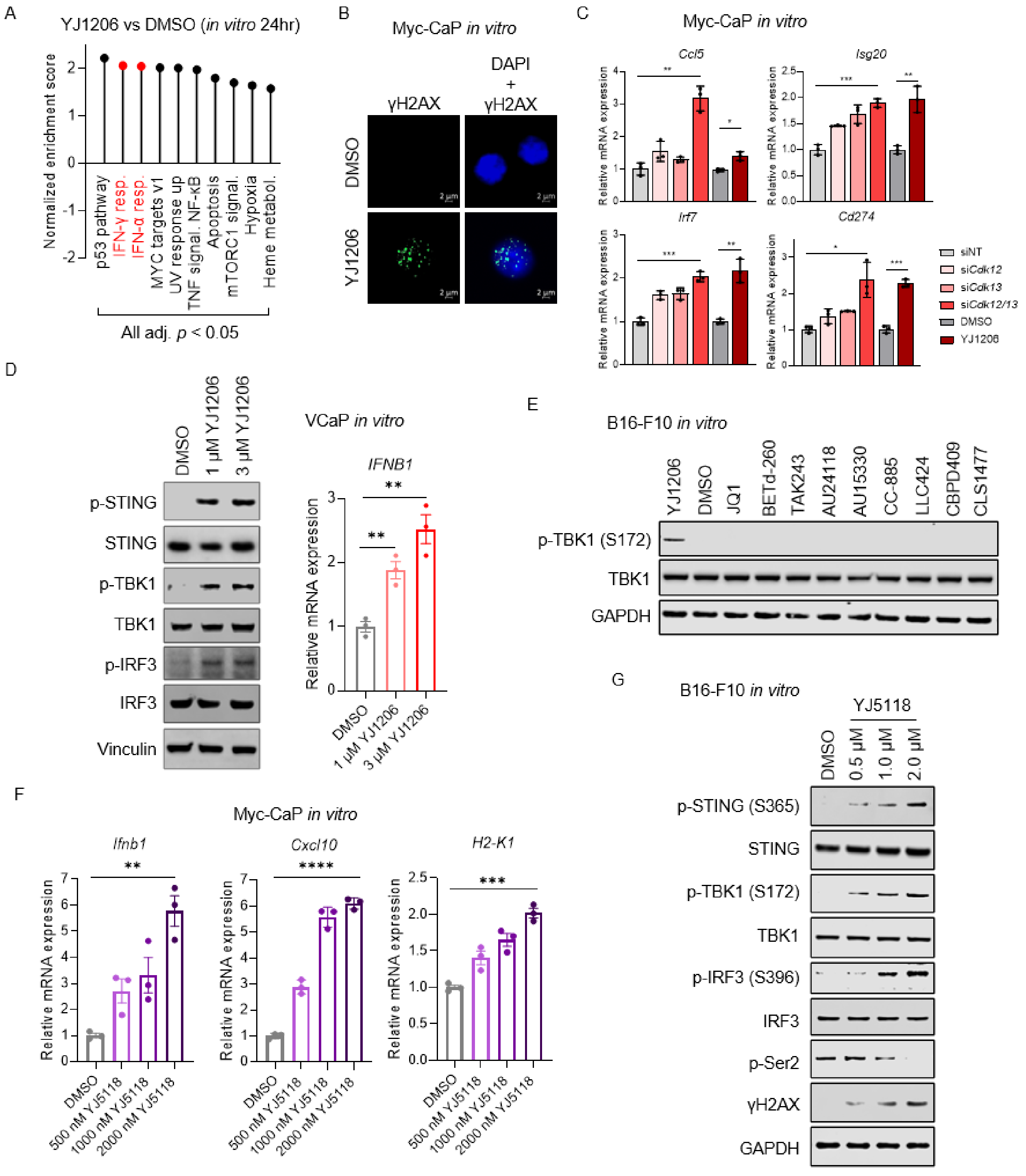

Supplemental Figure S3: A, The top 10 pathways enriched by GSEA, utilizing the MSigDB Hallmark database, in Myc-CaP cells treated with YJ1206 at 1 μM for 24 hours. **B**, Immunofluorescence images of γH2AX (green) in Myc-CaP cells treated with YJ1206 for 4 hours.

Scale bar: 2 μm. **C**, Analysis of *Ccl5*, *Isg20*, *Irf7*, and *Cd274* expression by RT-qPCR in Myc-CaP cells treated with YJ1206 at 1 μM for 15 hours, or siRNAs targeting *Cdk12* and/or *Cdk13*. Non-targeting siRNA was used as control. **D**, Immunoblot of the indicated proteins (left) or RT-qPCR measuring *IFNB1* expression (right) in VCaP cells treated with YJ1206 at the indicated concentrations for 4 hours (left) or 6 hours (right). **E**, Immunoblot of the indicated proteins in B16-F10 cells treated with the indicated compounds at 1 μM for 4 hours. **F**, RT-qPCR assessing expression of the indicated genes in Myc-CaP cells treated with YJ5118 at the indicated concentrations for 6 hours (left and middle) or 15 hours (right). **G**, Immunoblot of the indicated proteins in B16-F10 cells treated with YJ5118 at the indicated concentrations for 4 hours.

Data are displayed as mean ± S.D. in **C**, **D**, and **F**. Statistics in **C**, **D**, and **F** were acquired by two-tailed t test. **p*<0.05, ***p*<0.01, ****p*<0.001, *****p*<0.0001. Bonferroni correction was applied for multiple comparisons.

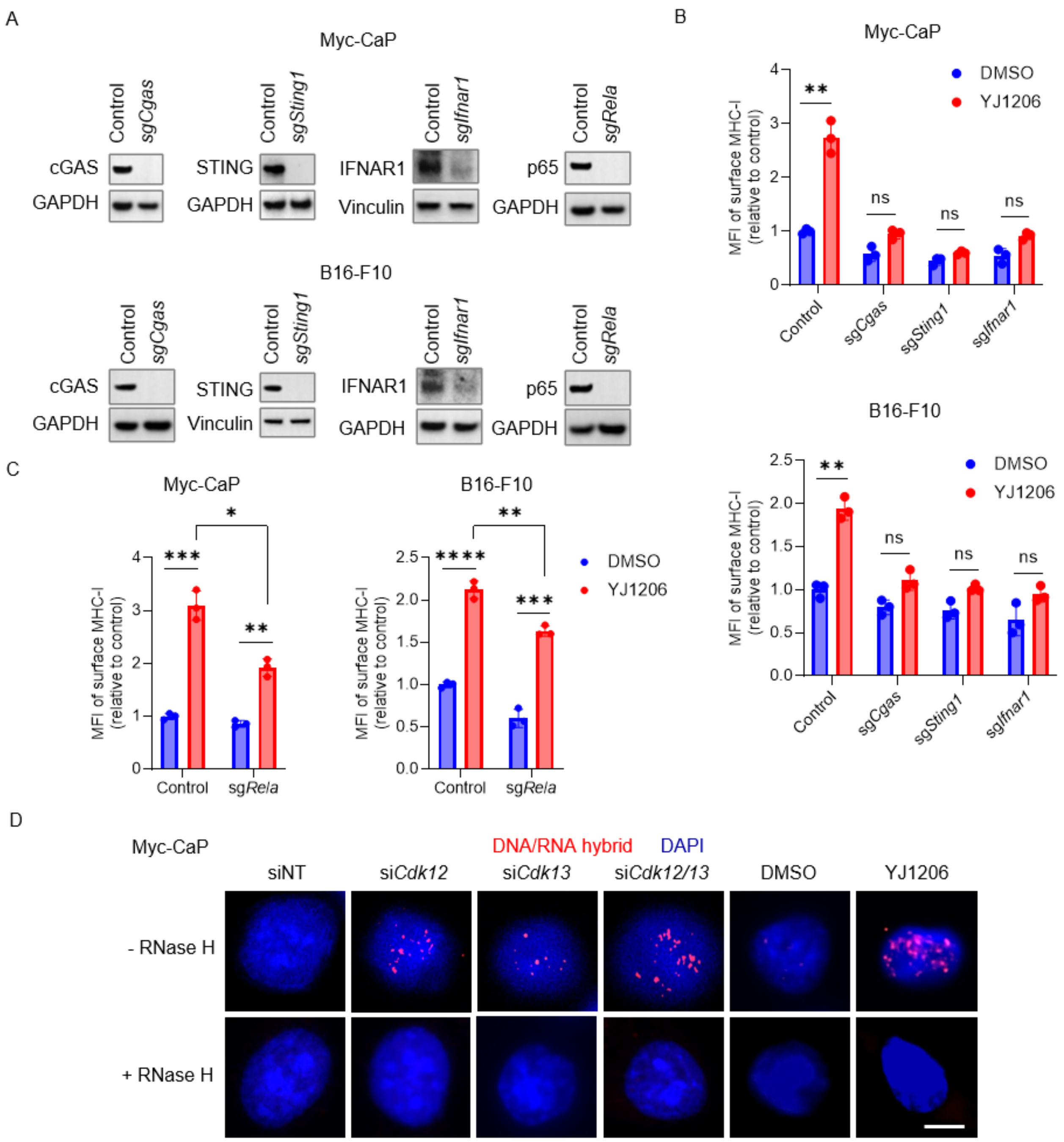

Supplemental Figure S4: A, Immunoblot of the indicated proteins in Myc-CaP or B16-F10 cells receiving the indicated sgRNAs. **B–C**, Flow cytometry measuring surface expression of MHC-I in the indicated cells receiving the specified sgRNAs and treated with YJ1206 at 1 μM for 15 hours. Data are displayed as mean ± S.D. Statistics were acquired by two-tailed t test. *p<0.05, **p<0.01,

***$p<0.001$, ****$p<0.0001$. ns: not significant. Bonferroni correction was applied for multiple comparisons. **D**, Representative images of immunofluorescence DNA/RNA hybrid (red) staining in Myc-CaP cells treated with 1 μM YJ1206 for 4 hours or siRNA targeting *Cdk12* and/or *Cdk13*, with/without RNase H. Quantification of the data is shown in Figure 2G. Scale bar: 5 μm.

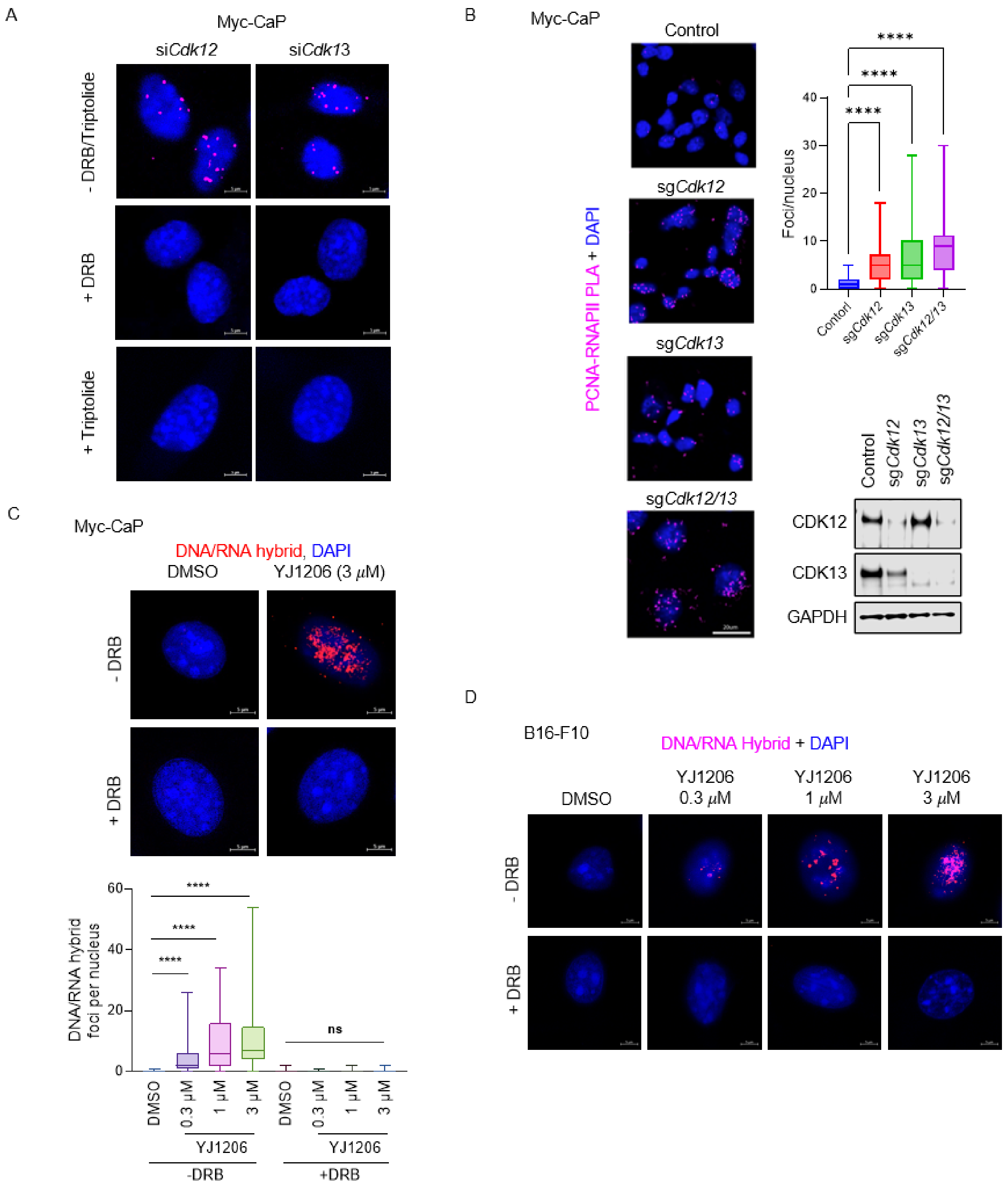

Supplemental Figure S5: A, Representative images of immunofluorescence assessing PCNA-RNAPII PLA foci in Myc-CaP cells treated with siRNA targeting *Cdk12* or *Cdk13* with or without

DRB or triptolide treatment. **B**, Representative images (left) or quantification (right, top) of immunofluorescence assessing PCNA-RNAPII PLA foci in Myc-CaP cells with or without sgRNA treatment depleting *Cdk12* and/or *Cdk13*. Right, bottom: Immunoblots of the indicated proteins in Myc-CaP cells with or without sgRNA treatment depleting *Cdk12* and/or *Cdk13*. Non-targeting sgRNA was used as control. Scale bar: 20 μm. **C**, Representative images (top) or quantification (bottom) of DNA/RNA hybrid (red) staining in Myc-CaP cells treated with YJ1206 at 3 μM for 4 hours, with or without DRB treatment. Scale bar: 5 μm. **D**, Immunofluorescence images of DNA/RNA hybrid staining with YJ1206 treatment at the indicated concentrations for 4 hours with/without DRB (75 μM, 200 min) in B16-F10 cells.

Scale bar: 5 μm in **A**, **C**, and **D**. Data in **B–C** are presented as box-and-whisker plots, with the median (center line), 25th–75th percentiles (box), and minimum to maximum values (whiskers), in **B** with 90 cells per data point and in **C** with 100 cells per data point. ****$p<0.0001$ by two-tailed t test. ns: not significant.

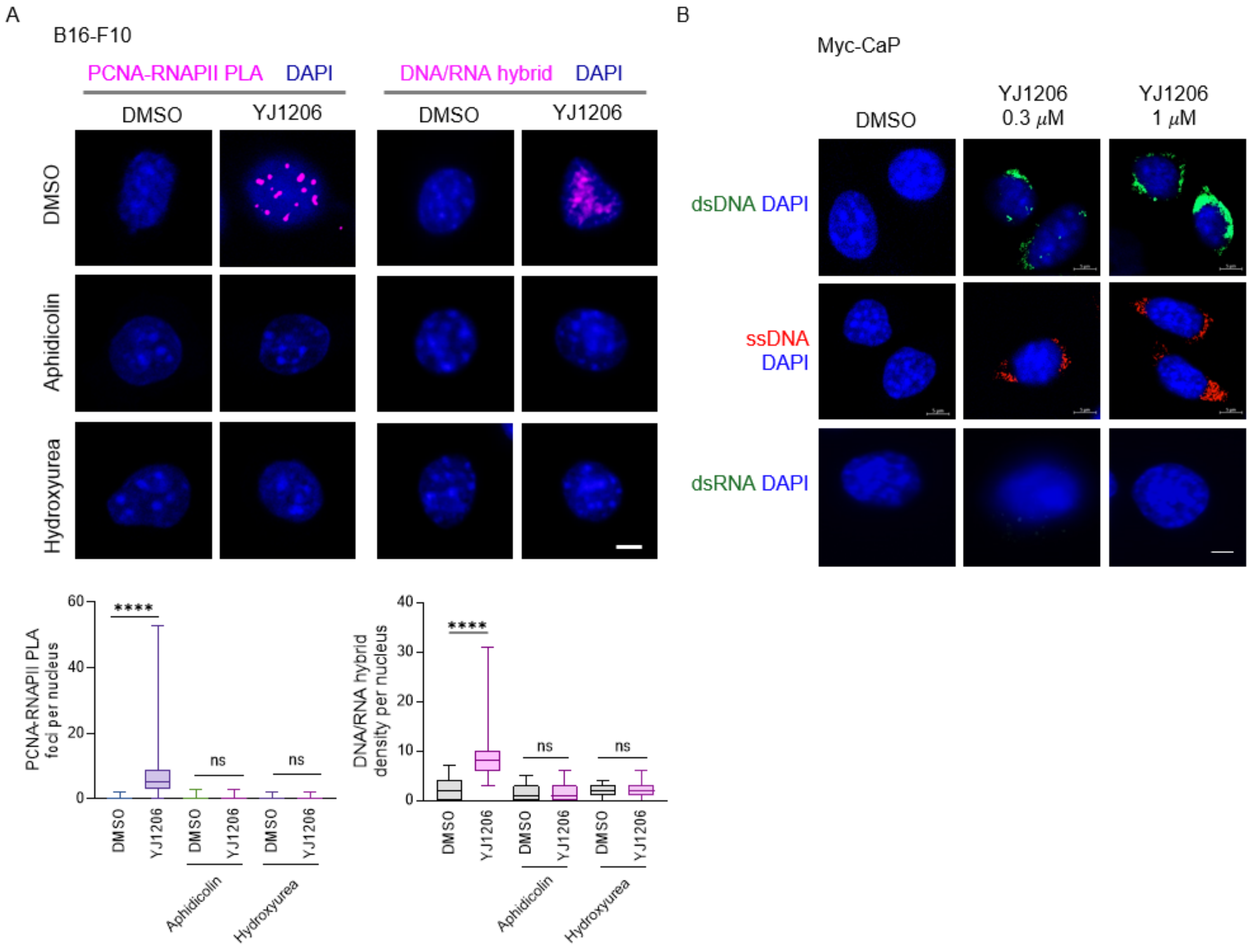

Supplemental Figure S6: A, Representative images (top) and quantification (bottom) of immunofluorescence analysis assessing PCNA–RNAPII proximity ligation assay (PLA) signals or DNA/RNA hybrid foci in B16-F10 cells treated with 1 μM aphidicolin or 1 mM hydroxyurea for 15 hours, followed by 3 μM YJ1206 for an additional 4 hours. Data are displayed as violin plots with 31-123 cells per data point. Statistics were acquired by two-tailed t test. ****p<0.0001. ns: not significant. Bonferroni correction was applied for multiple comparisons. Scale bar: 5 μm. Data are presented as box-and-whisker plots, with the median (center line), 25th–75th percentiles (box), and minimum to maximum values (whiskers). **B**, Representative images of immunofluorescence detecting dsDNA (top), ssDNA (middle), and dsRNA (bottom) in Myc-CaP cells with or without YJ1206 treatment at the indicated concentrations for 4 hours. Scale bar: 5 μm.

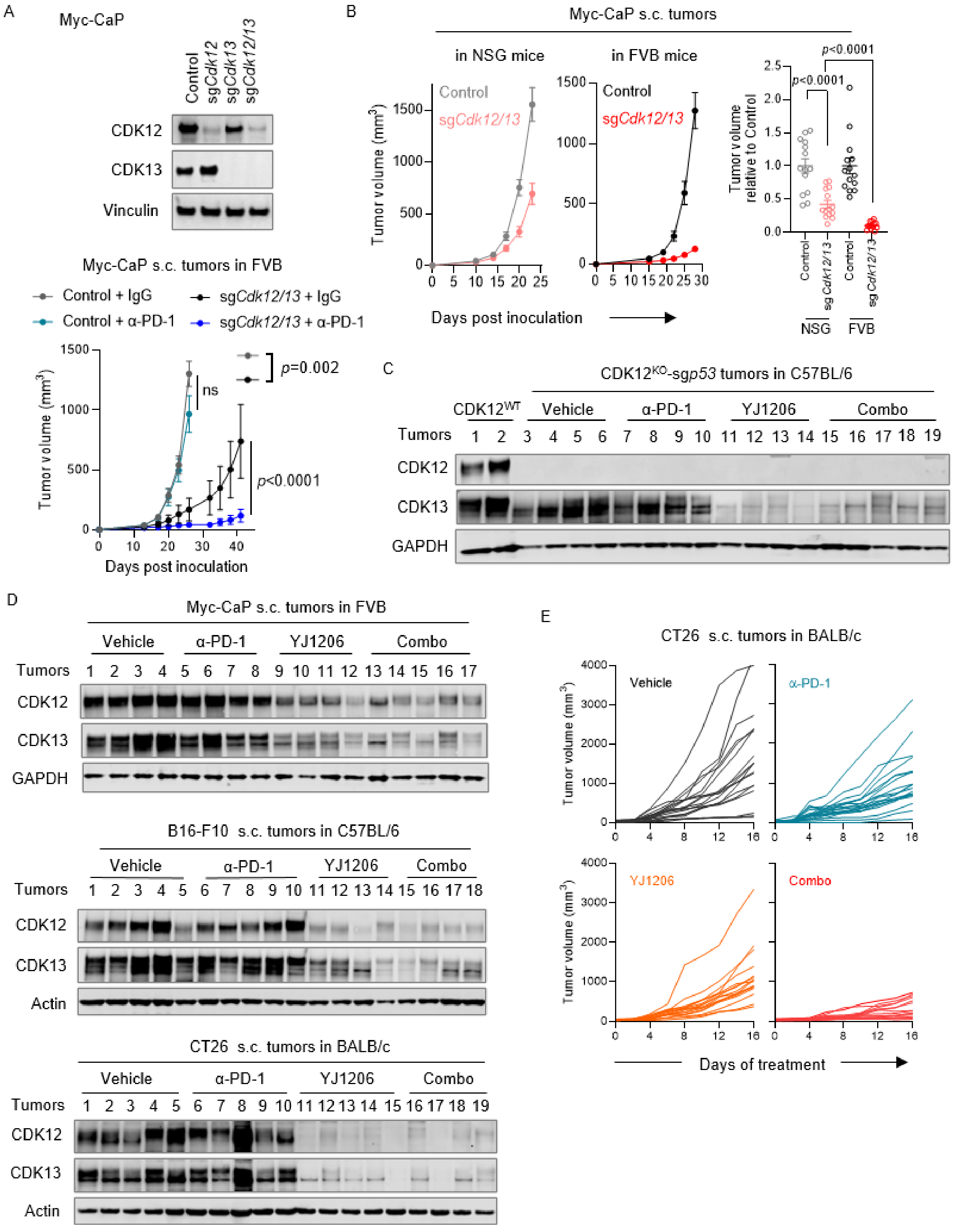
A
Myc-CaP
Control
sgCdk12
sgCdk13
sgCdk12/13
CDK12
CDK13
Vinculin
B
Myc-CaP s.c. tumors
in NSG mice
in FVB mice
Control
sgCdk12/13
Tumor volume (mm3)
Days post inoculation
Tumor volume relative to Control
p<0.0001
p<0.0001
NSG
FVB
Myc-CaP s.c. tumors in FVB
Control + IgG
Control + α-PD-1
sgCdk12/13 + IgG
sgCdk12/13 + α-PD-1
ns
p=0.002
p<0.0001
Tumor volume (mm3)
Days post inoculation
C
CDK12KO-sgp53 tumors in C57BL/6
CDK12WT
Vehicle
α-PD-1
YJ1206
Combo
Tumors
CDK12
CDK13
GAPDH
D
Myc-CaP s.c. tumors in FVB
Vehicle
α-PD-1
YJ1206
Combo
Tumors
CDK12
CDK13
GAPDH
B16-F10 s.c. tumors in C57BL/6
Vehicle
α-PD-1
YJ1206
Combo
Tumors
CDK12
CDK13
Actin
CT26 s.c. tumors in BALB/c
Vehicle
α-PD-1
YJ1206
Combo
Tumors
CDK12
CDK13
Actin
E
CT26 s.c. tumors in BALB/c
Vehicle
α-PD-1
YJ1206
Combo
Tumor volume (mm3)
Days of treatment

Supplemental Figure S7: A, Top: Immunoblot of indicated proteins for Myc-CaP cells with or without *Cdk12*, *Cdk13*, or *Cdk12/13* depletion. Bottom: Data from Figure 4A, replotted to show volumes of s.c. tumors established from control or sg*Cdk12/13* Myc-CaP cells, in FVB mice treated with IgG or anti-PD-1 (α-PD-1). ns: not significant. **B**, Volumes of s.c. tumors established from control or sg*Cdk12/13* Myc-CaP cells, in NSG (NOD.Cg-*Prkdc*^{scid} *Il2rg*^{tm1Wjl}/SzJ) or FVB mice (*n*=6–7 mice per group). **C**, Immunoblot of indicated proteins for *Cdk12*^{KO}-sg*p53* tumors from C57BL/6 mice treated with vehicle, α-PD-1, YJ1206, or the combination (combo) of α-PD-1 and YJ1206. **D**, Immunoblot of indicated proteins for the indicated tumors from the indicated mice treated with vehicle, α-PD-1, YJ1206, or the combination (combo) of α-PD-1 and YJ1206. **E**, Data from Figure 4D (right), replotted to show Individual growth curves of the indicated tumors in mice treated with the specified agents. YJ1206 was administered orally at a dose of 100 mg/kg, three times per week, and anti-PD-1 was administered intraperitoneally at a dose of 200 μg/mouse Q3d.

Data in **A**–**B** are displayed as mean ± S.E.M. Statistics in **A** were acquired by two-way ANOVA, and in **B** by two-tailed t test. Bonferroni correction was applied for multiple comparisons.

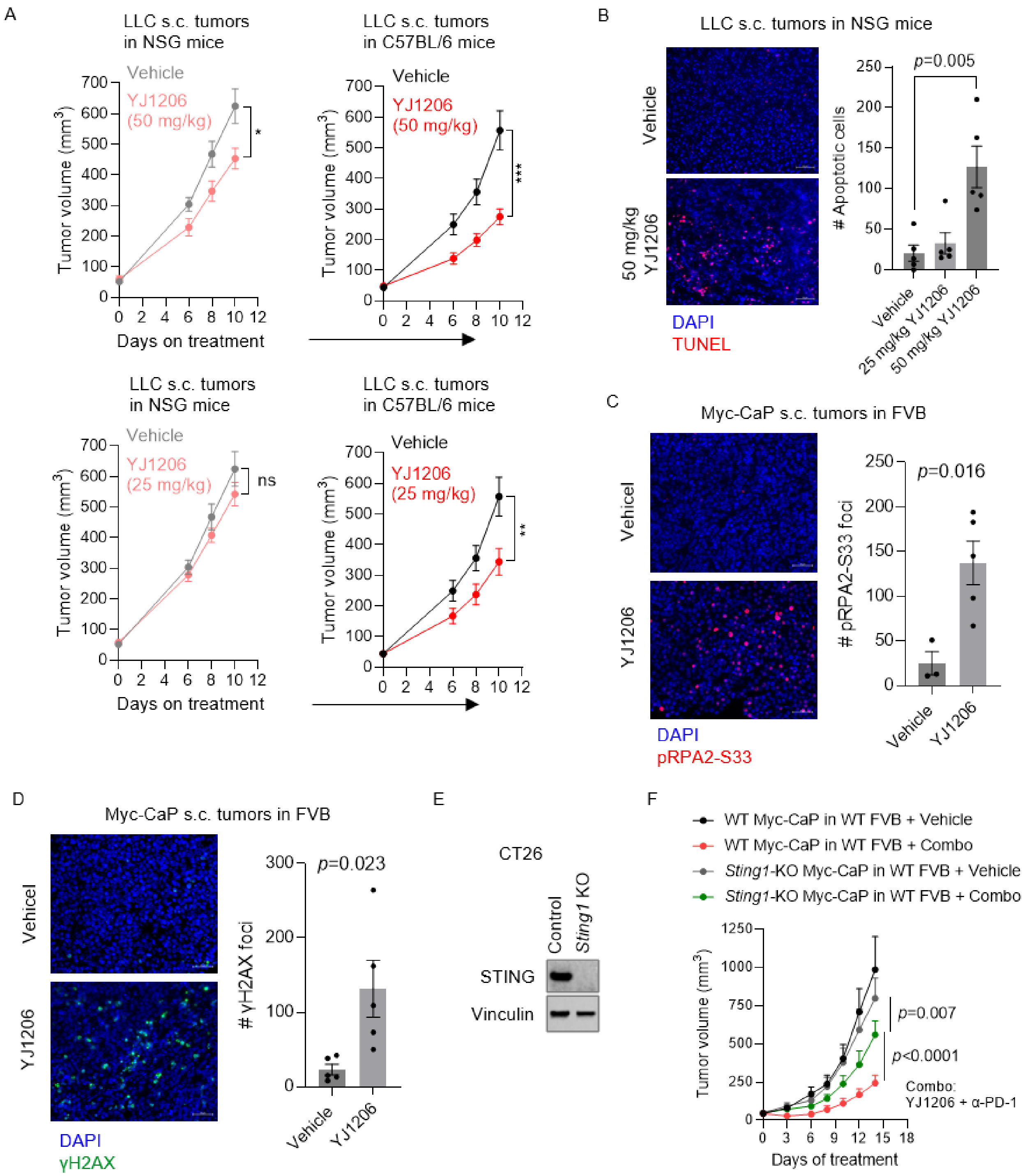

Supplemental Figure S8: A, Volumes of s.c. tumors established from LLC cells, in NSG or C57BL/6 mice, treated with the indicated doses of YJ1206. **B**, Representative images (left) or quantification (right) of TUNEL assay measuring apoptotic cells in LLC s.c. tumors from NSG mice

treated as in (**A**). Scale bar: 50 µm. *n* = 5 tumors per group. **C–D**, Representative images (left) or quantification (right) of immunofluorescence assessing pRPA2-S33 (**C**) or γH2AX (**D**) foci in Myc-CaP s.c. tumors from FVB mice treated with YJ1206. Scale bar: 50 µm. YJ1206 was administered orally at a dose of 100 mg/kg, three times per week. *n* = 3-5 tumors per group. **E**, Immunoblot of the indicated proteins in CT26 cells with or without *Sting1* (encoding STING) knockout (KO). **F**, Tumor growth curves of s.c. tumors derived from Myc-CaP cells with or without *Sting1* KO, in FVB mice treated with the specified agents (*n* = 4-5 mice per group). YJ1206 was administered orally at a dose of 100 mg/kg, three times per week, and anti-PD-1 was administered intraperitoneally at a dose of 200 µg/mouse Q3d.

Statistics were acquired by two-tailed t test in **A–D** and by two-way ANOVA in **F**. $*p<0.05$, $**p<0.01$, $***p<0.001$. ns: not significant.

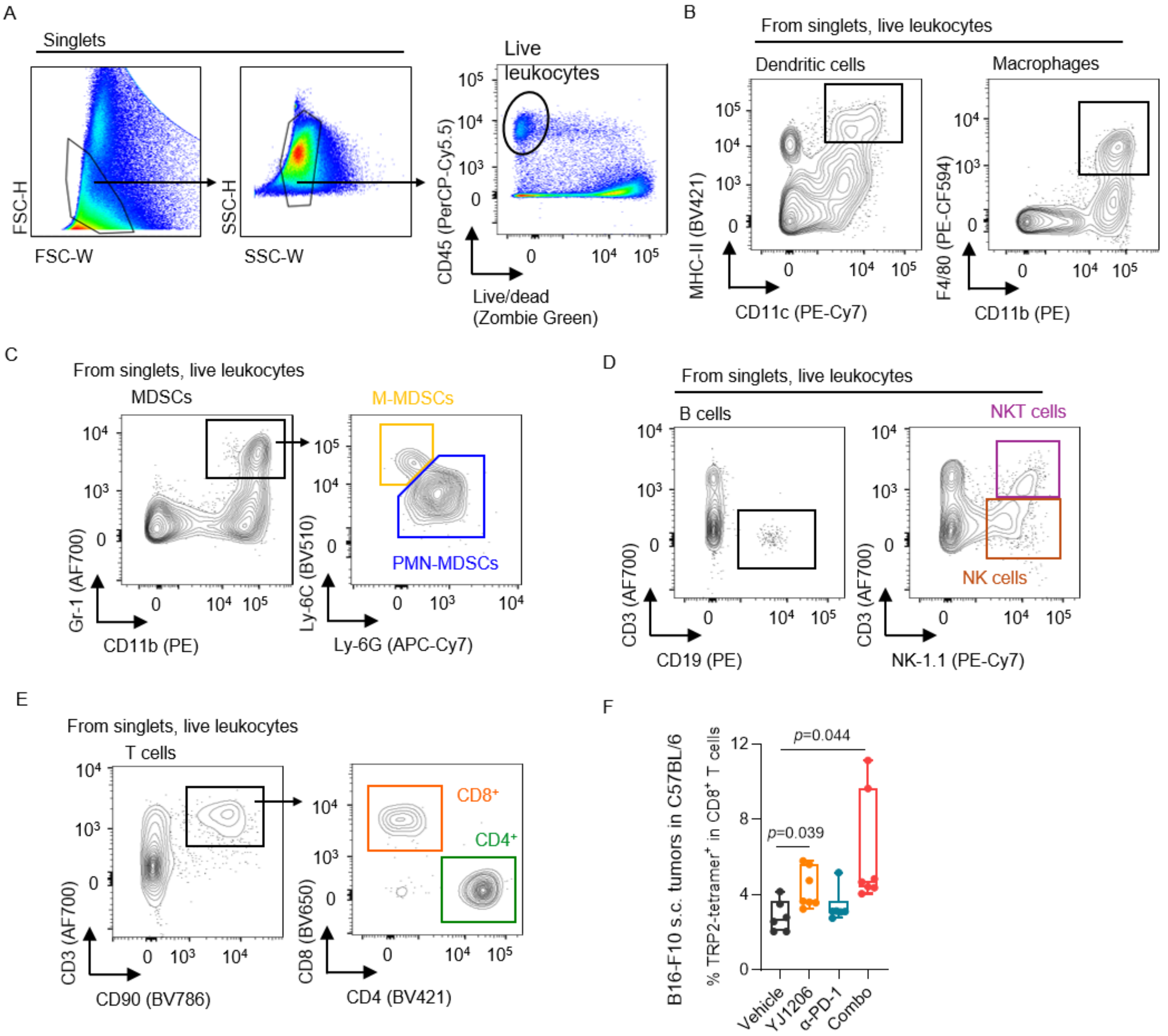

Supplemental Figure S9: A–E, Gating strategy for the indicated intratumoral cell population in flow cytometry. DCs: dendritic cells. MDSCs: myeloid-derived suppressor cells. PMN-MDSCs: polymorphonuclear myeloid-derived suppressor cells. M-MDSCs: monocytic myeloid-derived suppressor cells. **F**, Quantification of flow cytometry measuring the fraction of CD8$^+$ T cells specific to TRP2 in total CD8$^+$ T cells in the indicated tumor models treated with vehicle, α-PD-1, YJ1206, or the combination (combo) of α-PD-1 and YJ1206 (n = 6-7 mice per group). YJ1206 was administered orally at a dose of 100 mg/kg, three times per week, and anti-PD-1 was administered intraperitoneally at a dose of 200 μg/mouse Q3d. Statistics were acquired by two-

tailed t test with Bonferroni correction. Data are presented as box-and-whisker plots, with the median (center line), 25th–75th percentiles (box), and minimum to maximum values (whiskers).

sgRNA	Target sequence
sg*Cdk12* #1	CTTCCTCCTGAGCTACCAGG
sg*Cdk12* #2	TGTCTTCTTTCTCCATAACG
sg*Cdk13* #1	CTTCCTGGCTGCCCCCGGCG
sg*Cdk13* #2	ATGCTTCTACACCTACCAAG
sg*Sting1* #1	GAAGGCCAAACATCCAACTG
sg*Sting1* #2	AGTATGACCAGGCCAGCCCG
sg*Ifnar1*	GCTTCTAAACGTACTTCTGG
sg*Cgas*	TGATAAGAAGTGTTACAGCA
sg*Rela*	GCCCAGACCGCAGTATCCAT
Nontargeting control	ATTGTTCGACCGTCTACGGG

Supplemental Table S3: sgRNAs used in this study.

Name	Sequence
*Ifnb1*_Rev	AGGACATCTCCCACGTCAAT
*Ifnb1*_Fwd	TGAACTCCACCAGCAGACAG
*Cxcl10*_Rev	CCTATGGCCCTCATTCTCAC
*Cxcl10*_Fwd	CGTCATTTTCTGCCTCATCC
*H2-K1*_Rev	TGTGGAAGGGAAGACAGAGC
*H2-K1*_Fwd	CCCTGTGAGCCTATGGACTC
*Cd274*_Rev	TGATCTGAAGGGCAGCATTTC
*Cd274*_Fwd	GCTCCAAAGGACTTGTACGTG
*Actb*_Rev	TACGACCAGAGGCATACAGG
*Actb*_Fwd	GGCCAACCGTGAAAAGATGA

Supplemental Table S4: RT-qPCR primers used in this study.

Supplementary Tables S1 (Excel file): clinical and demographic characteristics of the MI-ONCOSEQ cohort.

Supplementary Tables S2 (Excel file): *CDK12* and *CDK13* expression and survival data from ICB cohorts, downloaded from the KM-plotter.
