# Supplementary material for: CDK12/13 inactivation triggers STING-mediated antitumor immunity in preclinical models
Source: J Clin Invest. 2025 Jul 22;135(18):e193745. doi: 10.1172/JCI193745 (PMC12435847; doi:10.1172/JCI193745)

# Figure 2

## Fig 2B

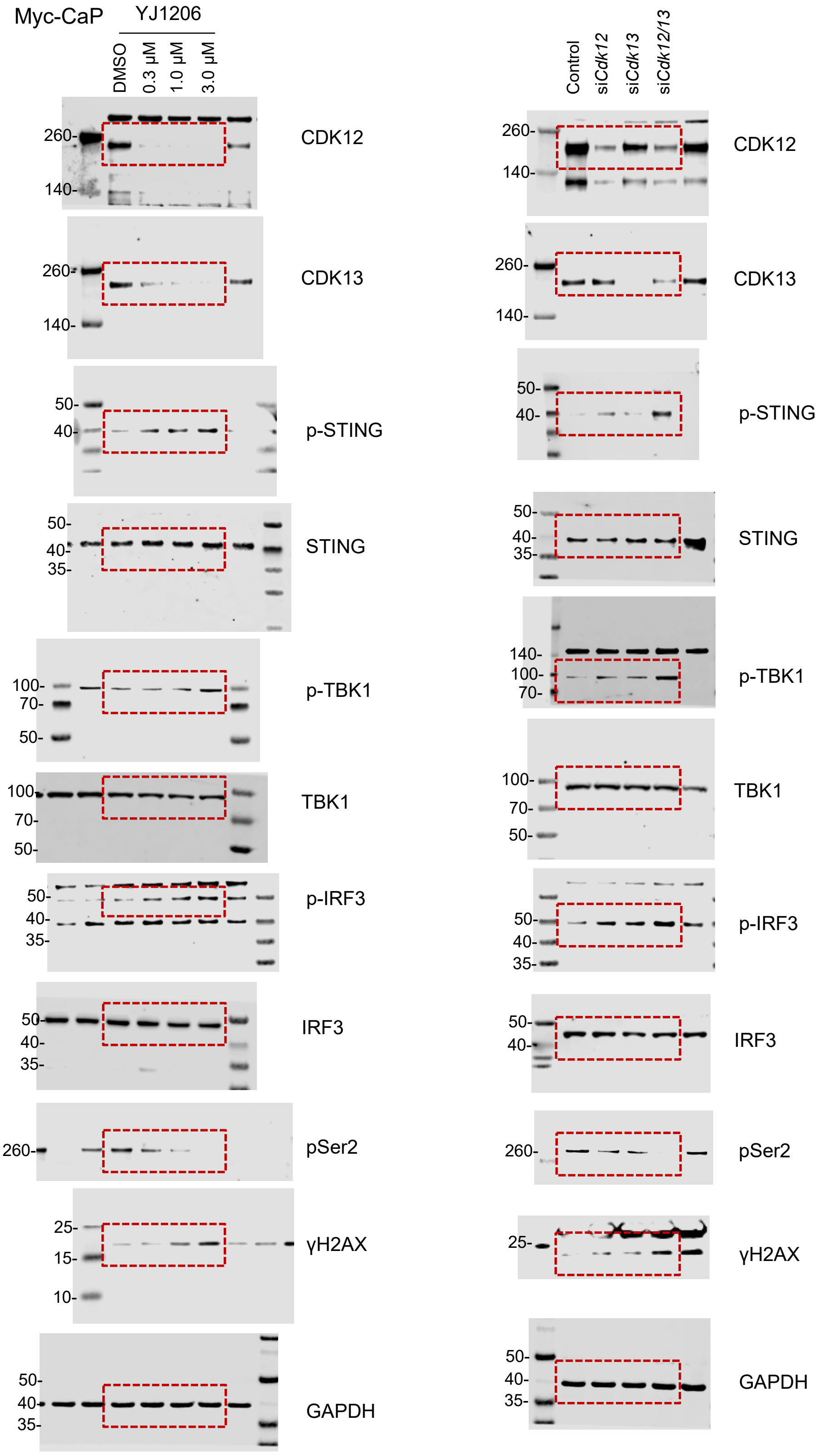

## Fig 2D

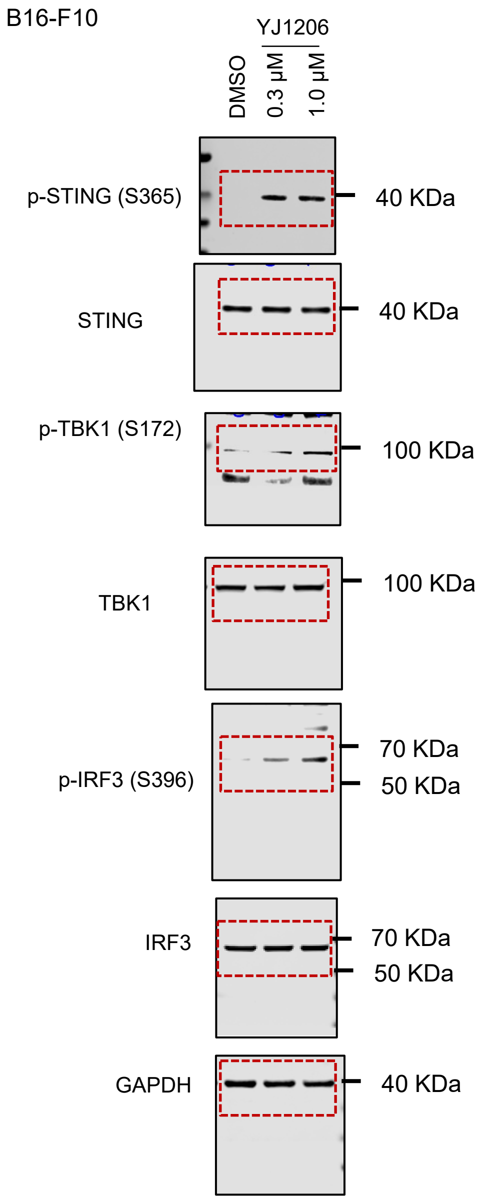

B16-F10
YJ1206
DMSO
0.3 μM
1.0 μM
p-STING (S365)
40 KDa
STING
40 KDa
p-TBK1 (S172)
100 KDa
TBK1
100 KDa
p-IRF3 (S396)
70 KDa
50 KDa
IRF3
70 KDa
50 KDa
GAPDH
40 KDa


## Fig 3G

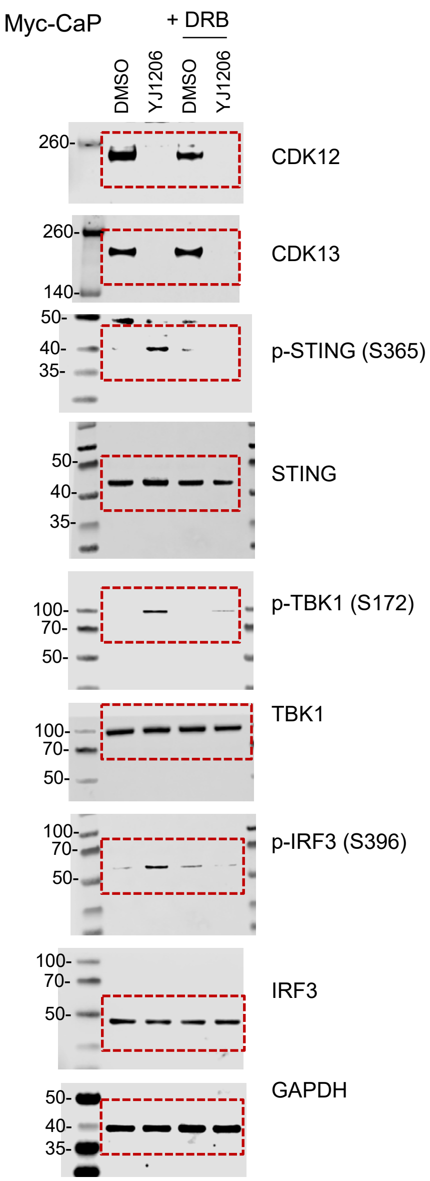

Myc-CaP
+ DRB
DMSO
YJ1206
DMSO
YJ1206
260-
CDK12
260-
140-
CDK13
50-
40-
35-
p-STING (S365)
50-
40-
35-
STING
100-
70-
50-
p-TBK1 (S172)
100-
70-
50-
TBK1
100-
70-
50-
p-IRF3 (S396)
100-
70-
50-
IRF3
50-
40-
35-
GAPDH

**Figure S3D**

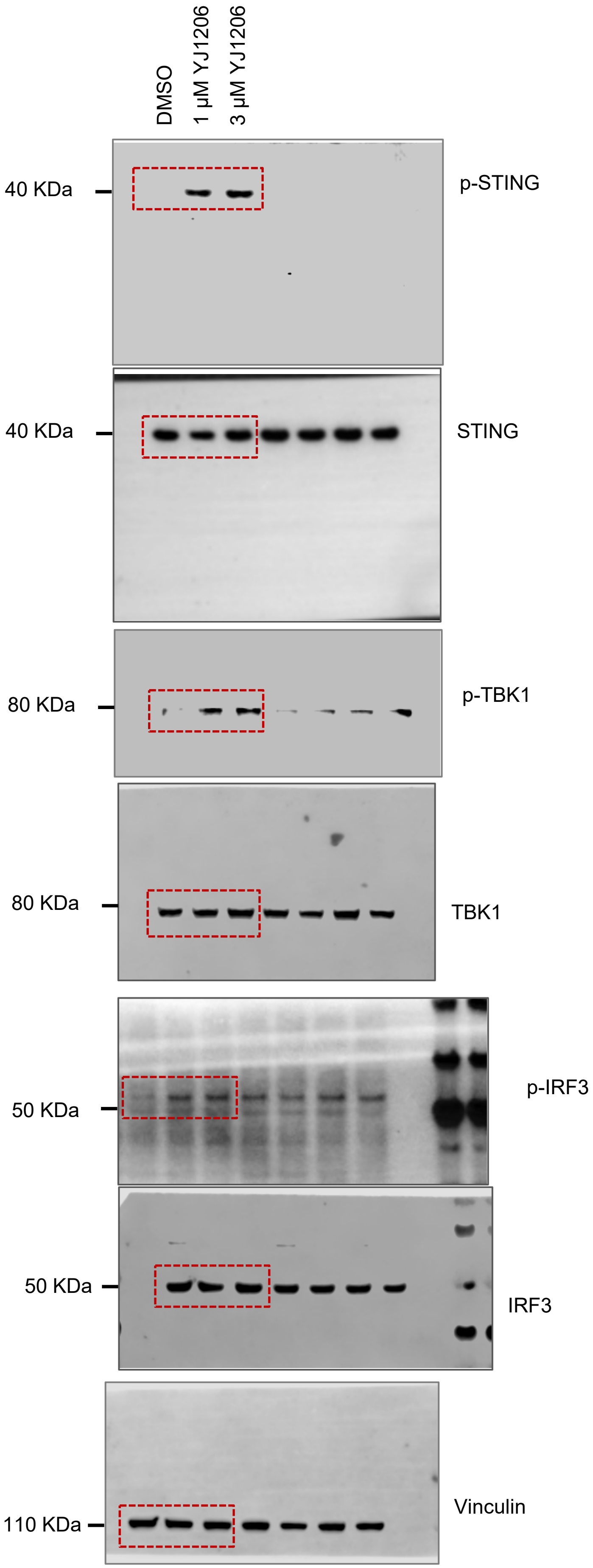
DMSO
1 µM YJ1206
3 µM YJ1206
40 KDa
p-STING
40 KDa
STING
80 KDa
p-TBK1
80 KDa
TBK1
50 KDa
p-IRF3
50 KDa
IRF3
110 KDa
Vinculin

## Figure S3

### Fig S3E

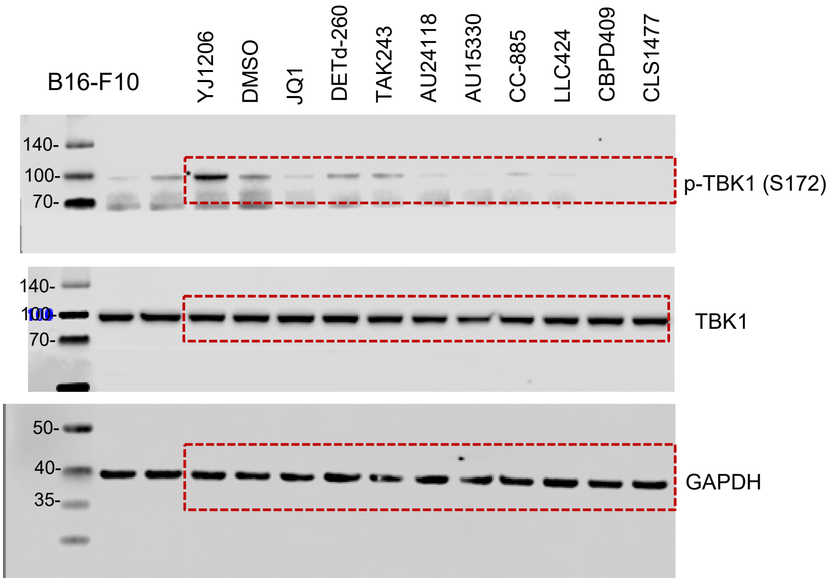


### Figure S3G

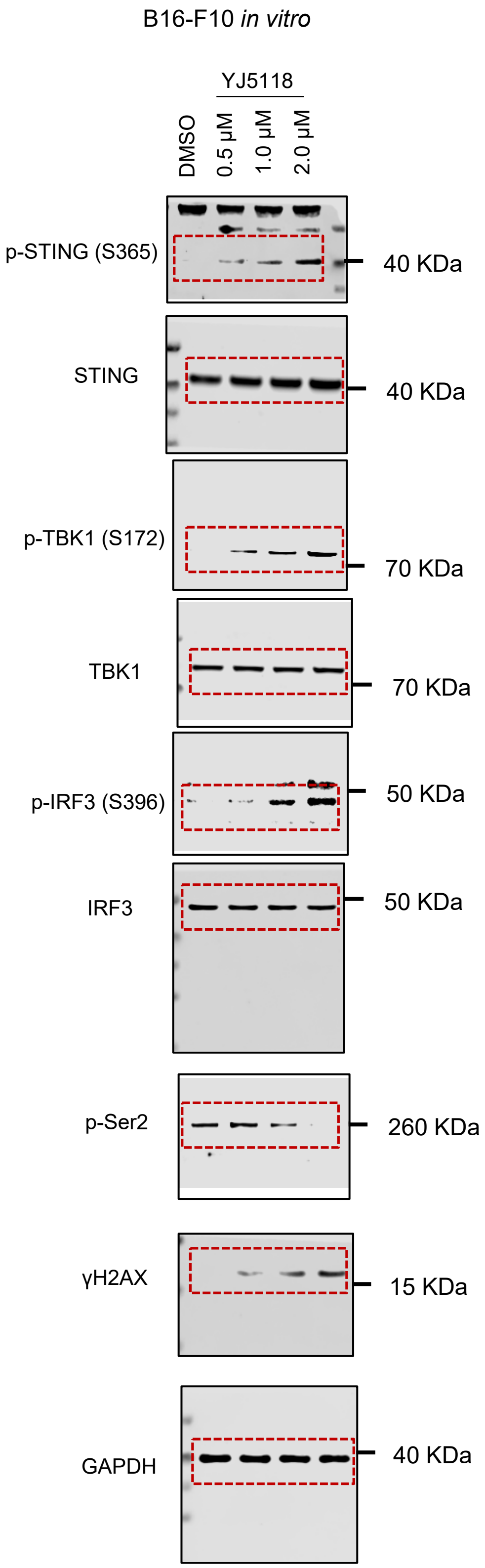

## Figure S4A

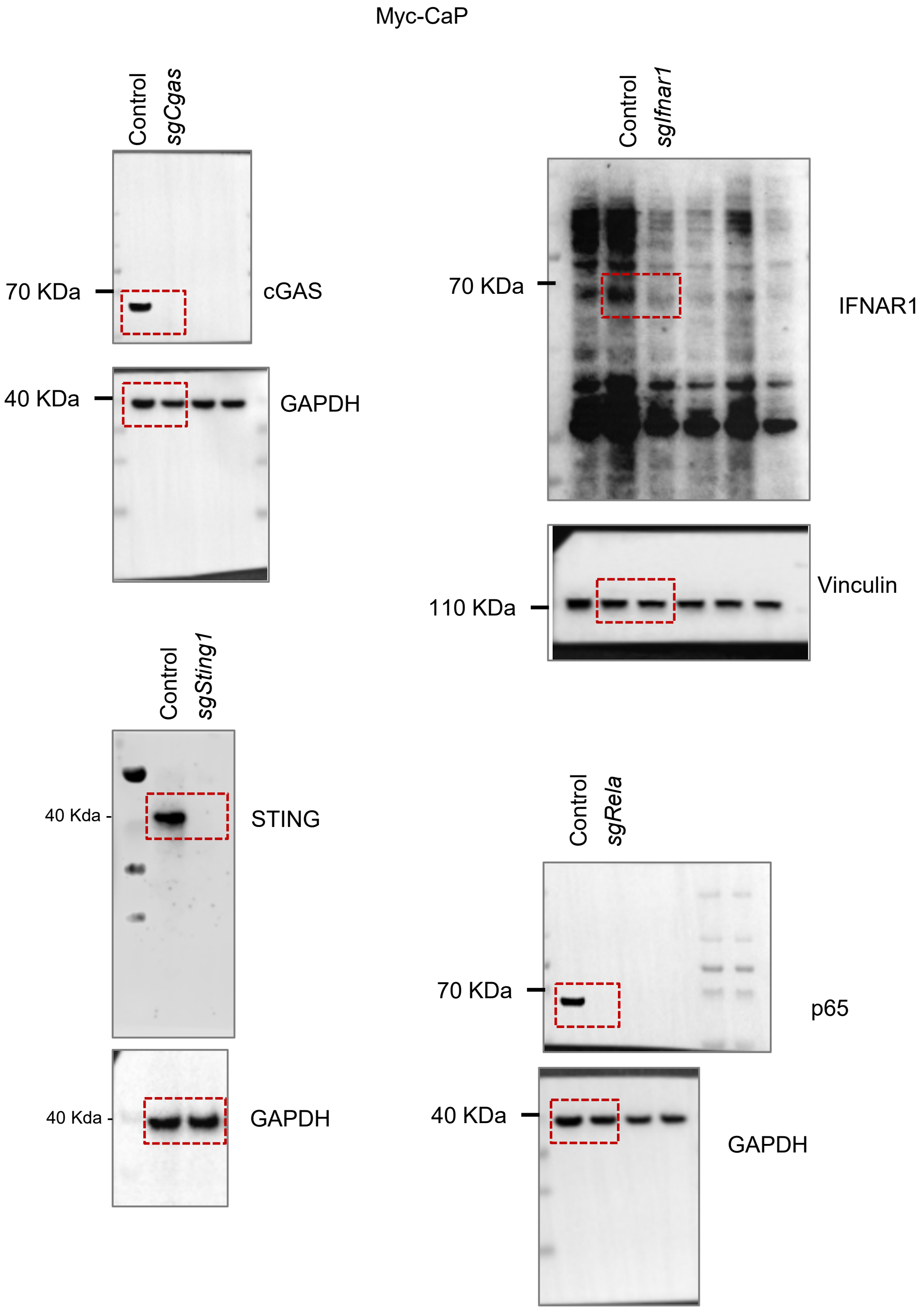
Myc-CaP
Control
sgCgas
70 KDa
cGAS
40 KDa
GAPDH
Control
sgIfnar1
70 KDa
IFNAR1
110 KDa
Vinculin
Control
sgSting1
40 Kda
STING
40 Kda
GAPDH
Control
sgRela
70 KDa
p65
40 KDa
GAPDH

# Figure S4A

B16-F10

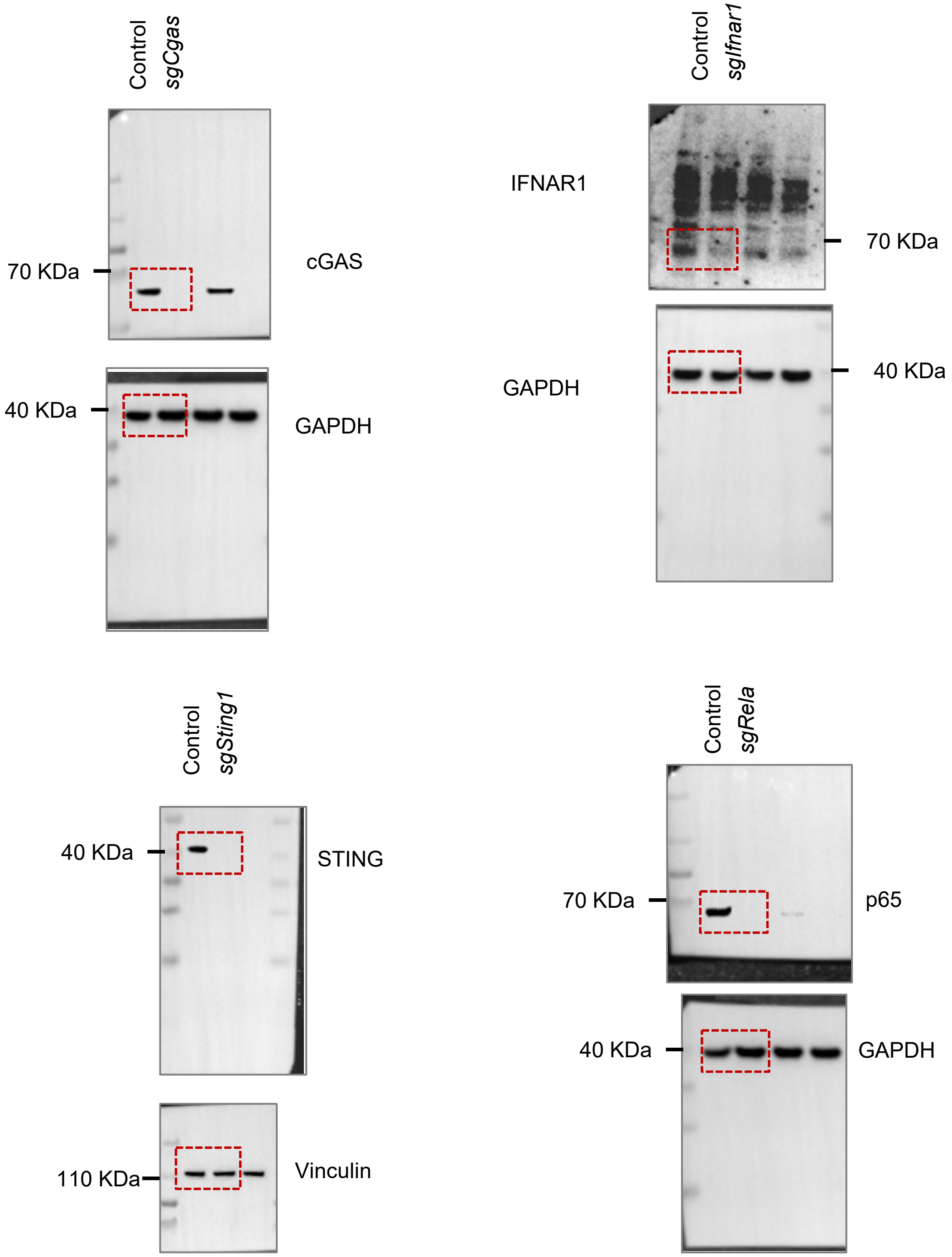

## Fig S5B

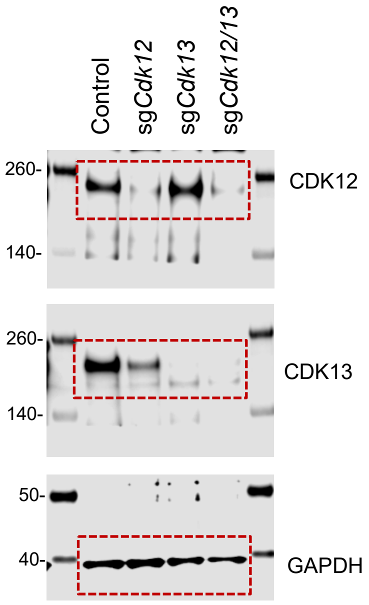


## Figure S7A

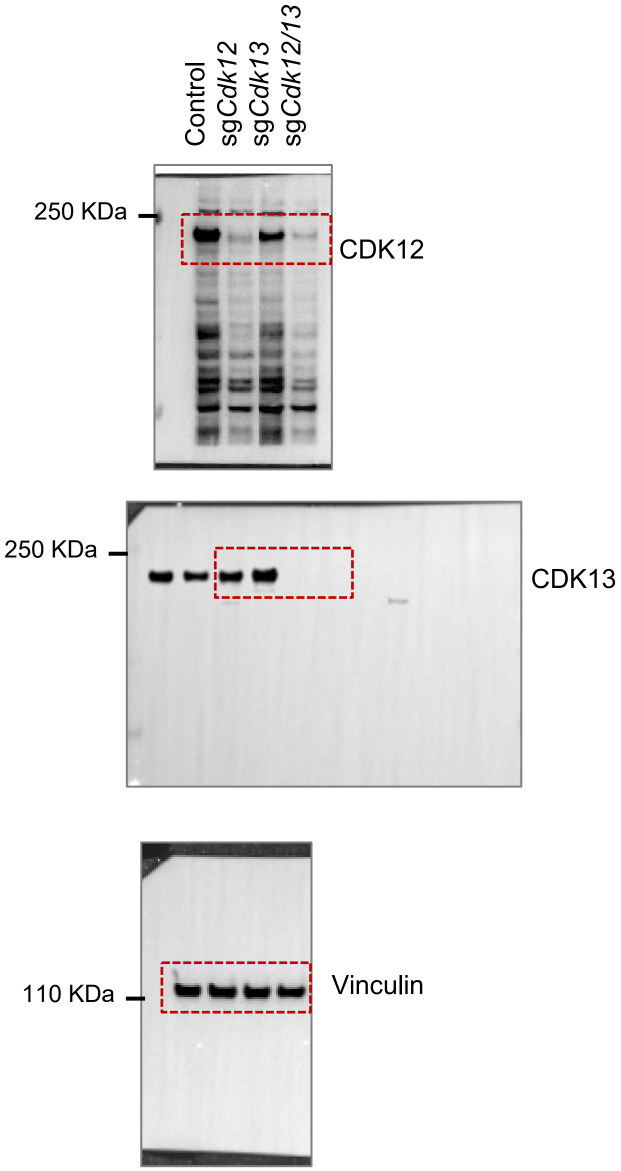

**Figure S7**

**Fig S7C**

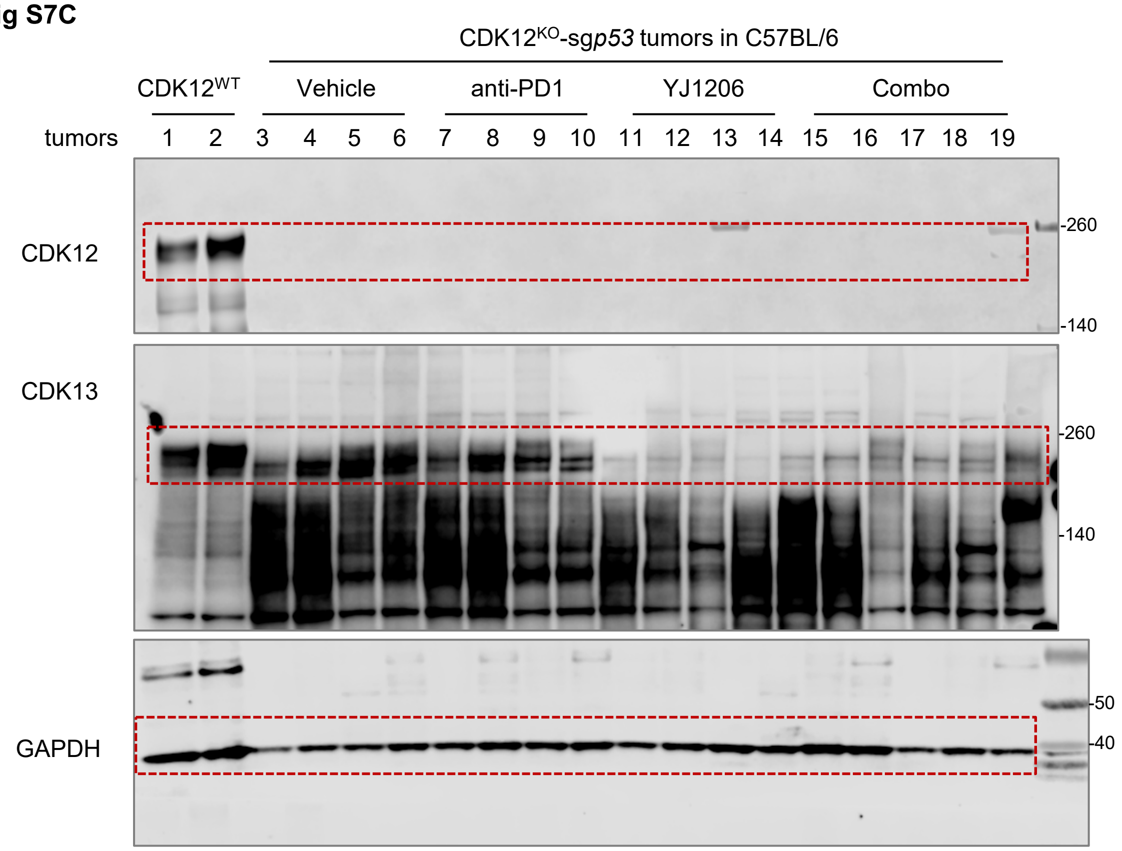


**Fig S7D**

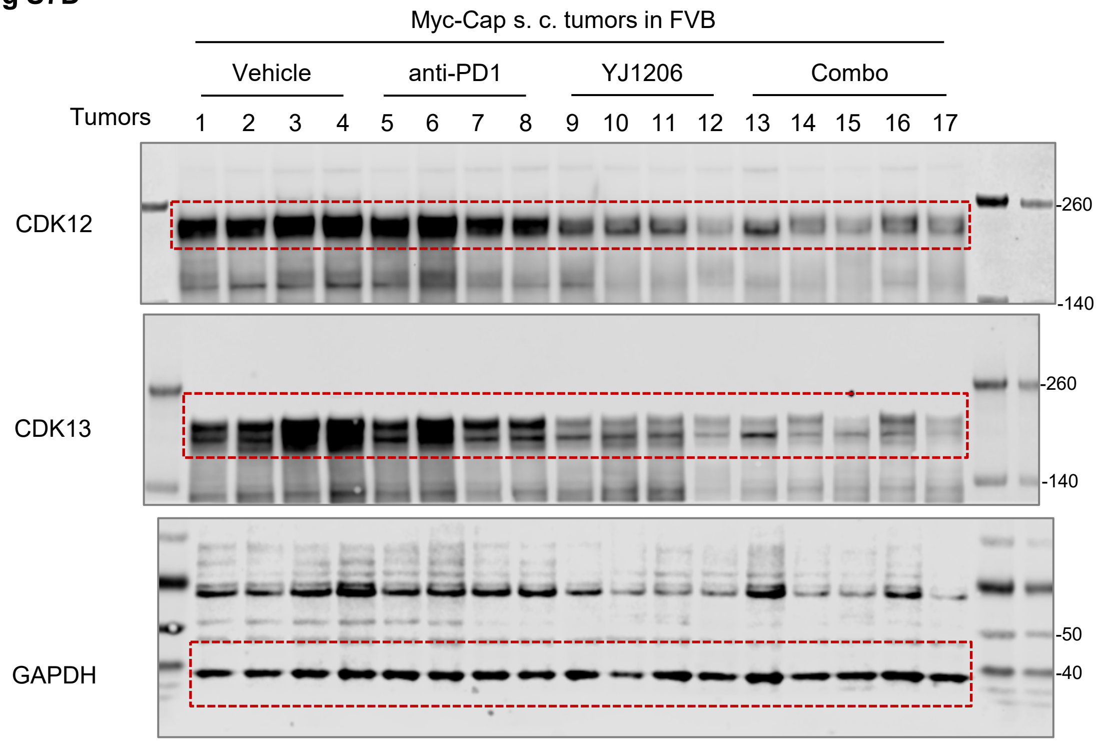

**Fig S7D**

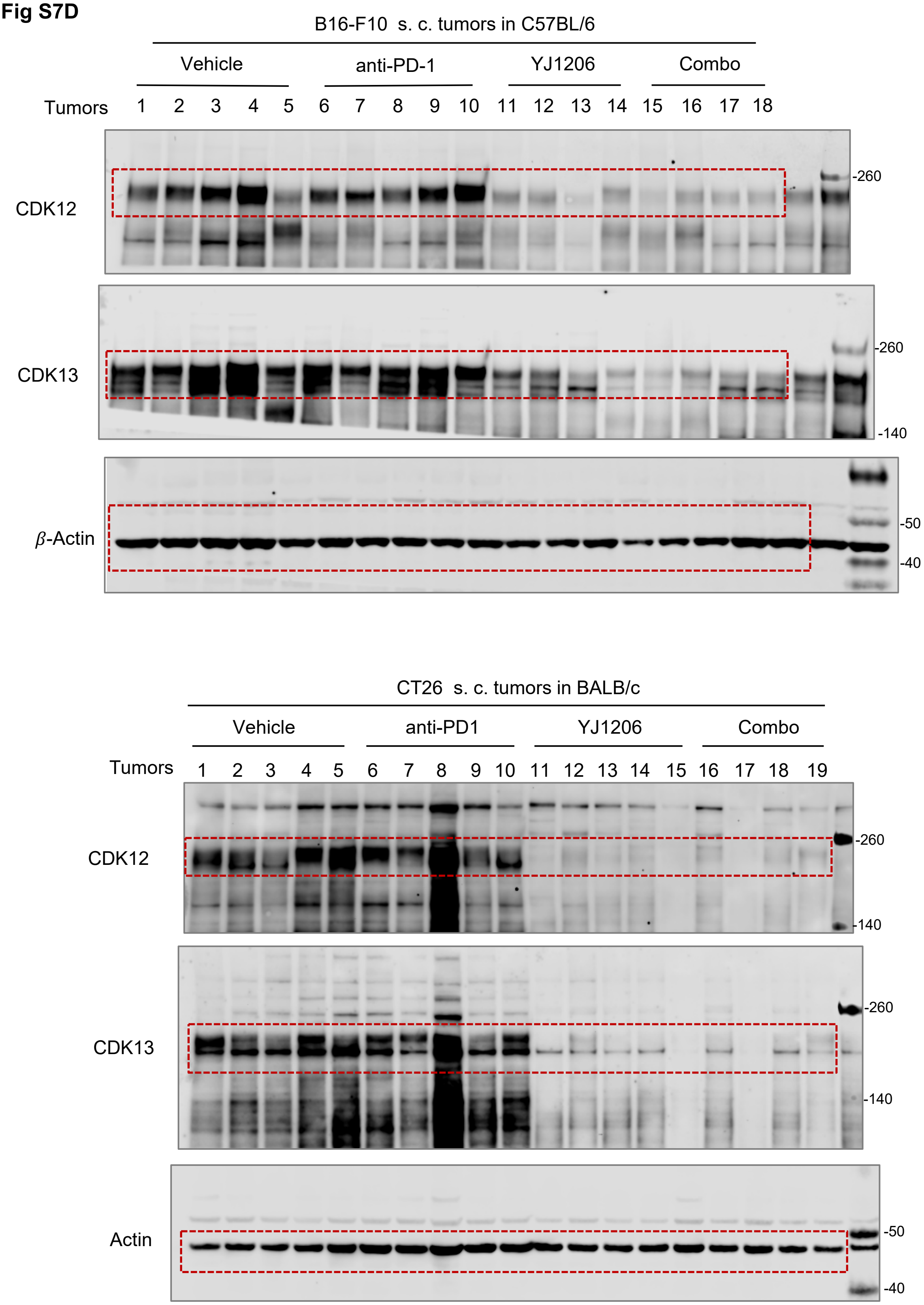
B16-F10 s. c. tumors in C57BL/6
Vehicle
anti-PD-1
YJ1206
Combo
Tumors 1 2 3 4 5 6 7 8 9 10 11 12 13 14 15 16 17 18
CDK12
-260
CDK13
-260
-140
β-Actin
-50
-40
CT26 s. c. tumors in BALB/c
Vehicle
anti-PD1
YJ1206
Combo
Tumors 1 2 3 4 5 6 7 8 9 10 11 12 13 14 15 16 17 18 19
CDK12
-260
-140
CDK13
-260
-140
Actin
-50
-40

**Figure S8E**

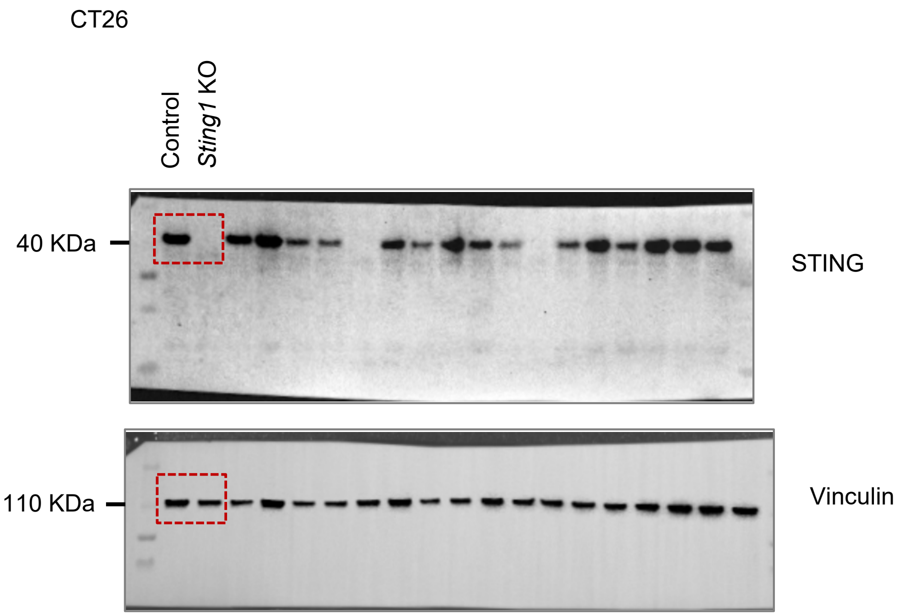

Supplement: Unedited blot and gel images [file jci-135-193745-s094.pdf]
